# Mitochondrial fragmentation affects neither the sensitivity to TNFα-induced apoptosis of *Brucella*-infected cells nor the intracellular replication of the bacteria

**DOI:** 10.1038/s41598-018-23483-3

**Published:** 2018-03-26

**Authors:** Elodie Lobet, Kevin Willemart, Noëlle Ninane, Catherine Demazy, Jaroslaw Sedzicki, Christophe Lelubre, Xavier De Bolle, Patricia Renard, Martine Raes, Christoph Dehio, Jean-Jacques Letesson, Thierry Arnould

**Affiliations:** 1 Laboratory of Biochemistry and Cell Biology (URBC, Unité de Recherche en Biologie Cellulaire)-NARILIS (Namur Research Institute for Life Sciences), University of Namur, Rue de Bruxelles 61, 5000 Namur, Belgium; 2 Microorganisms Biology Research Unit (URBM, Unité de Recherche en Biologie des Microorganismes)-NARILIS (Namur Research Institute for Life Sciences), University of Namur, Rue de Bruxelles 61, 5000 Namur, Belgium; 3 Biozentrum, University of Basel, Klingelbergstrasse 50/70, 4056 Basel, Switzerland; 4 Laboratory of Experimental Medicine (ULB 222 Unit), Medicine Faculty, Université Libre de Bruxelles, CHU de Charleroi, Rue de Gozée 706, 6110 Montigny-le-Tilleul, Belgium

## Abstract

Mitochondria are complex organelles that participate in many cellular functions, ranging from ATP production to immune responses against viruses and bacteria. This integration of a plethora of functions within a single organelle makes mitochondria a very attractive target to manipulate for intracellular pathogens. We characterised the crosstalk that exists between *Brucella abortus*, the causative agent of brucellosis, and the mitochondria of infected cells. *Brucella* replicates in a compartment derived from the endoplasmic reticulum (ER) and modulates ER functionality by activating the unfolded protein response. However, the impact of *Brucella* on the mitochondrial population of infected cells still requires a systematic study. We observed physical contacts between *Brucella* containing vacuoles and mitochondria. We also found that *B. abortus* replication is independent of mitochondrial oxidative phosphorylation and that mitochondrial reactive oxygen species do not participate to the control of *B. abortus* infection *in vitro*. We demonstrated that *B. abortus* and *B. melitensis* induce a drastic mitochondrial fragmentation at 48 hours post-infection in different cell types, including myeloid and non-myeloid cells. This fragmentation is DRP1-independent and might be caused by a deficit of mitochondrial fusion. However, mitochondrial fragmentation does not change neither *Brucella* replication efficiency, nor the susceptibility of infected cells to TNFα-induced apoptosis.

## Introduction

Mitochondria are essential organelles that evolved from an endosymbiotic α-proteobacterium of the *Rickettsia* genus^1^. Despite their subsequent evolution, mitochondria still share many similarities with prokaryotic cells, such as a double membrane, the capacity to produce ATP through oxidative phosphorylation (OXPHOS) and the presence of their own genome and bacterial-type ribosomes^2^. Mitochondria are highly dynamic organelles that continuously adapt their morphology and move to specific cellular sub-compartments, using different components of the cytoskeleton, to respond to cellular needs^3^.

The mitochondrial morphology is controlled by the balance between mitochondrial fission and fusion and is mediated by large GTPases related to the dynamin superfamily. On the one hand, fusion occurs as a two-step mechanism: a fusion of the outer mitochondrial membrane (OMM), mediated by the homo-/hetero-dimerisation of mitofusin1/2 (MFN1/2), is followed by the formation of homodimers of optic atrophy 1 (OPA1), which leads to fusion of the inner mitochondrial membrane (IMM)^3^. On the other hand, fission requires the recruitment of dynamin-related protein 1 (DRP1) to the OMM, where it assembles to form a constriction ring that leads to fission. Four different receptors for DRP1, located in the mitochondrial outer membrane, have been identified so far in mammalian cells: mitochondrial fission 1 (FIS1), mitochondrial fission factor (MFF) and mitochondrial dynamics protein of 49 and 51 kDa (MID49 and MID51). Fission occurs where the endoplasmic reticulum (ER) marks the localization of DRP1 recruitment in collaboration with elements of the actin cytoskeleton^3^. Mitochondrial dynamics and the various functions and roles of this organelle are interconnected^4^. Indeed, according to the cell type and functional status, the organelle structure will vary from an interconnected and branched network that promotes exchanges between the mitochondrial fragments, to individual rounded entities that facilitate the movement, segregation and degradation of impaired mitochondria, thereby preventing the accumulation and propagation of mitochondrial dysfunction^5,6^.

In addition to being the main ATP producers of the cell, through OXPHOS, mitochondria also fulfil many other functions, such as contributing to lipid, amino acid and nucleotide syntheses and catabolism, integration of pro- and anti-apoptotic signals, control of calcium homeostasis and redox signalling. Mitochondria are also a cell signalling hub through sensing of Pathogen-Associated Molecular Patterns (PAMPs) and by initiating signalling pathways such as apoptosis and innate immune responses^7–9^. The concentration of these various functions in one single organelle makes mitochondria a target of choice for intracellular pathogens. Several bacteria (e.g. *Listeria monocytogenes* and *Vibrio cholera*) and viruses (e.g. Hepatitis C and Epstein-Barr) are reported to manipulate mitochondria during infection^10–12^. In the present study, we analysed the effect of *Brucella abortus* on the biology of mitochondria of myeloid (RAW 264.7 macrophage) and non-myeloid (HeLa) cells.

*Brucella* spp. are Gram-negative, facultative, intracellular bacteria responsible for brucellosis, a worldwide zoonosis. Brucellosis leads to abortion and sterility in animals, whereas *Brucella* infection in humans causes undulating fever and articular, cardiac and neurological complications during the chronic phase of the infection^13^. Once inside the infected cell, *Brucella* is contained in vacuoles (BCV, for *Brucella-*containing vacuoles) that interact with and acquire the markers of different components of the endosomal pathway^14^. In most cell types, the BCV transiently interact with the lysosomes to reach, *in fine*, an ER-derived compartment where bacteria replicate massively^14^. The transition from the endosomal pathway to the ER is dependent on VirB, the type IV secretion system (T4SS) of *Brucella*^14^.

Considering the massive replication of *Brucella* in the ER, different groups have shown that the unfolded protein response (UPR), an ER stress response, is activated in *Brucella*-infected cells^15–17^. These observations suggest that the ER is under stress and that its functions are affected during *Brucella* infection. The ER and mitochondria are two organelles that interact both physically and functionally, and ER stress is known to modify mitochondrial functions^18,19^. It thus makes sense to analyse the impact of *Brucella* infection on the mitochondrial population of infected cells.

A very recent study demonstrated that *B. abortus* disrupts mitochondrial energy production by inducing a Warburg-like metabolic shift in human macrophages, which is associated with increased bacterial survival^20^. Furthermore, additional evidences suggest that other mitochondrial functions might be affected during *Brucella* infection. One transcriptomic study revealed the down-regulation of several nuclear genes encoding mitochondrial proteins in *B. melitensis*-infected RAW 264.7 macrophages at 4 h post-infection (PI)^21^. Mitochondrial ROS (mtROS) were also reported to participate in the production of interleukin-1β (IL-1β) in *B. abortus*-infected murine macrophages, in a process involving the NOD-Like Receptor family Pyrin domain containing 3 (NLRP3)^22^. In addition, BCV fractions obtained by subcellular fractionation of *B. abortus*-infected BHK-21 cells, were systematically contaminated by mitochondria and the immune-precipitation of mitochondria in those fractions lead to the loss of most of the BCVs^23^, suggesting the possibility of a physical interaction between the mitochondria and BCV.

In the present study, we highlighted the presence of intimate contacts between BCV and mitochondria, suggesting that these structures might physically interact. We show that *B. abortus* replication does not rely on mitochondrial OXPHOS and that mtROS do not participate in the control of *B. abortus* infection *in vitro*. However, we demonstrate that *Brucella* infection induces a strong fragmentation of the mitochondrial network in infected cells in a process that does not involve DRP1. This mitochondrial fragmentation might be caused by a deficit of fusion. Indeed, the abundance of MFN1 and MFN2 is dramatically reduced in mitochondria-enriched fractions prepared from infected cells, while the levels OPA1 remain unchanged. We further showed that *Brucella*-induced mitochondrial fragmentation does not affect the sensitivity of *Brucella*-infected cells to Tumour-Necrosis Factor α (TNFα)-induced apoptosis, nor does it impair replication of the bacteria in the host cells.

## Results

### *Brucella* and mitochondria might physically interact

**Figure 1:**
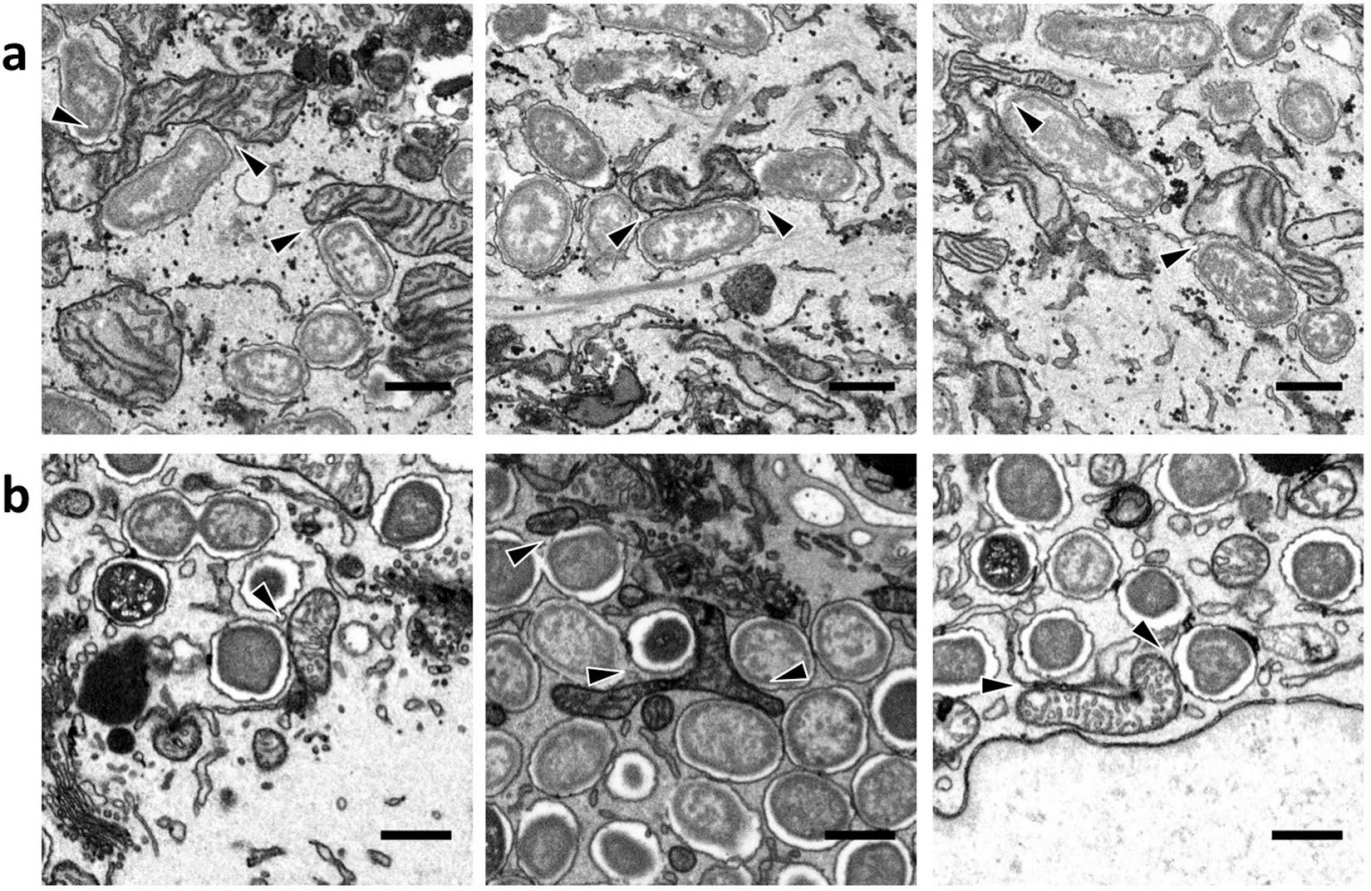
Mitochondria and BCV seem to interact physically during *Brucella abortus* infection (**a**). Electron microscopy analyses of *B. abortus* 2308 RFP-infected HeLa cells – 24 h PI. Scale bars represent 600 nm (**b**). Electron microscopy analyses of trophoblasts from *B. melitensis* 16M-infected BALB/c mice (10^5^ bacteria/mouse – intraperitoneal infection) – 5 d PI. Scale bars represent 600 nm.

First, we studied the potential interactions between *Brucella* and mitochondria by performing a transmission electron microscopy analysis of the ultrastructure of the infected cells. We observed, in many cases, close contacts between BCVs and mitochondria, both *in vitro*, in *B. abortus*-infected HeLa cells (24 h PI) (Fig. 1a), and *in vivo*, in trophoblasts from BALB/c mice intraperitoneally infected with *B. melitensis* (5 d PI) (Fig. 1b). These observations suggest that BCV might physically interact with mitochondria.

### Inhibition of mitochondrial respiration and modulation of mtROS content in host cells does not prevent *B. abortus* replication

**Figure 2:**
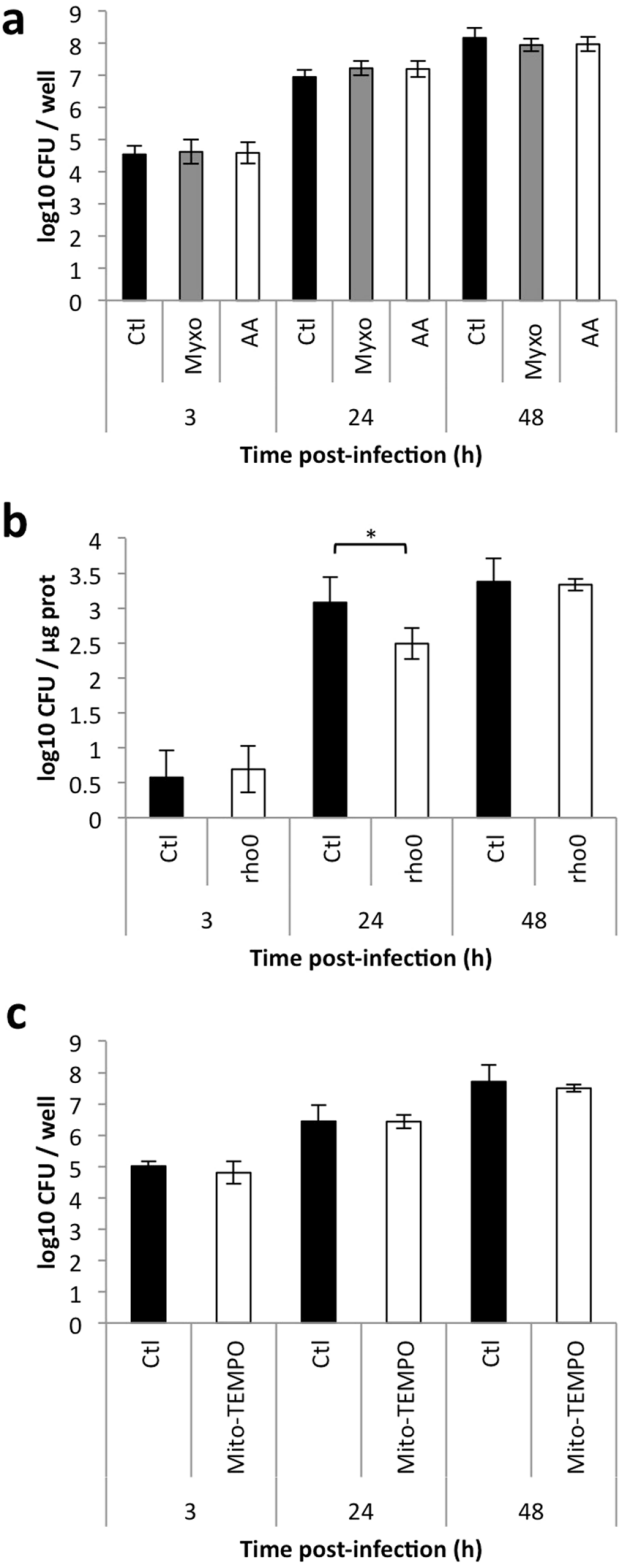
Mitochondrial dysfunction does not modulate *Brucella abortus* replication (**a**). CFU/well of RAW 264.7 macrophages preincubated for 6 h with or without (Ctl) 10 nM myxothiazol (Myxo) or 100 nM antimycin A (AA) and infected with *B. abortus* 2308 with or without the inhibitors. Results represent means ± SD for three independent experiments (n = 3). Statistical analysis: two-way ANOVA (**b**). CFU/µg of proteins of HeLa EB1 (Ctl) and rho^0^ infected with *B. abortus* 2308. Results represent means ± SD for three independent experiments (n = 3). Statistical analysis: two-way ANOVA. (*P < 0.05) (**c**). CFU/well of RAW 264.7 macrophages pre-incubated for 1 h with or without (Ctl) 500 µM Mito-TEMPO and infected with *B. abortus* 2308 with or without Mito-TEMPO. Results represent means ± SD for two independent experiments (n = 2). Statistical analysis: two-way ANOVA on ranks.

Given the possible interaction between BCVs and mitochondria, we addressed the question of the role of mitochondrial bioenergetics during *Brucella* infection by comparing the infection efficiency in two models of mitochondrial respiration dysfunction. The first model consists of RAW 264.7 macrophages incubated with inhibitors of complex 3 of the electron transport chain (ETC C3): antimycin A and myxothiazol. RAW 264.7 cells were pre-incubated with or without 10 nM myxothiazol or 100 nM antimycin A for 6 h to induce ATP depletion (a decrease of approximately 80 %) (Supp. Fig. S1a). Cells were then infected with *B. abortus* 2308, with or without inhibitors. Colony forming units (CFU) were analysed at several times post-infection (PI) to monitor *Brucella* replication. Inhibition of ETC C3 did not affect *Brucella* replication (Fig. 2a). Under the same conditions, we also used confocal microscopy to assess the percentages of cells infected by *B. abortus* 2308 mCherry and we did not observe any difference in the proportion of infected cells between the treated and untreated cells with ETC C3 (Supp. Fig. S1b).

We further investigated the relative importance of OXPHOS in *Brucella* infection using cells depleted in mitochondrial DNA (mtDNA) as host cells. The depletion of mtDNA triggers a complete loss of ATP production by OXPHOS^24^. HeLa rho^0^ cells, depleted in mtDNA by an ethidium bromide treatment, were compared with EB1 cells (cells generated from HeLa rho^0^ cells and recolonised with WT mtDNA)^24^. As shown in Supp. Fig. S1c, rho^0^ cells are completely depleted of mitochondrial genome-encoded mitochondrial proteins (such as the subunit I of the Cytochrome *c* Oxidase, COX I) but not of nuclear genome-encoded mitochondrial proteins (such as COX IV). Rho^0^ and EB1 HeLa cells were infected with *B. abortus* 2308 and CFU were counted at several time points PI. In this experiment, the number of CFU was normalised for protein content to account for the differences in the doubling time observed between these two cell lines^24^. As shown in Fig. 2b, *Brucella* replication was comparable in both cell lines, even if we detect a significant difference at 24 h PI. *Brucella* replication was comparable in both cell lines, even if we detect a significant difference at 24 h PI. This could suggest a difference in kinetics of replication but, at the end time point analysed (48 h PI), this effect is not observed anymore. Therefore, even if we cannot completely rule out that *Brucella* replication might be partially supported by mitochondrion-dependent ATP production, mitochondrial respiration is not required for *B. abortus* replication.

The mtROS are also crucial for elicitation of the immune response, by taking part in cell signalling and by a direct bactericidal effect^25^. We therefore analysed the putative impact of the mtROS content on *B. abortus* replication. Antimycin A inhibits the ETC C3, but it also triggers mtROS production^26^, as confirmed using flow cytometry. Our analysis of the superoxide anion radicals content in RAW 264.7 macrophages incubated with 100 nM antimycin A for different incubation times and then loaded with the MitoSOX probe (Supp. Fig. S1d) revealed a higher ROS production in cells incubated with antimycin A (48 h post-treatment). The presence of antimycin A did not change the numbers of CFU recovered from RAW 264.7 macrophages following infection with *B. abortus* (Fig. 2a), so we concluded that bacterial replication is not affected by mtROS production.

To support this conclusion, the effect of Mito-TEMPO, a specific mitochondrial-targeted superoxide scavenger^27^, was tested on bacterial replication (Fig. 2c**)**. We used a protocol previously reported to lower mtROS content^22^, namely a 1 h pre-incubation of RAW 264.7 macrophages with 500 µM Mito-TEMPO, and then infected the cells with *B. abortus* 2308 in the presence or not of the antioxidant. At several time points PI, the number of CFU was analysed, but we did not observe any effect of Mito-TEMPO on *B. abortus* replication (Fig. 2c). Taken together, these results suggest that mtROS do not participate in the control of *Brucella* infection, as neither an increase nor a decrease in the mtROS content affects *Brucella* replication.

### *Brucella* induces mitochondrial fragmentation in infected cells

**Figure 3:**
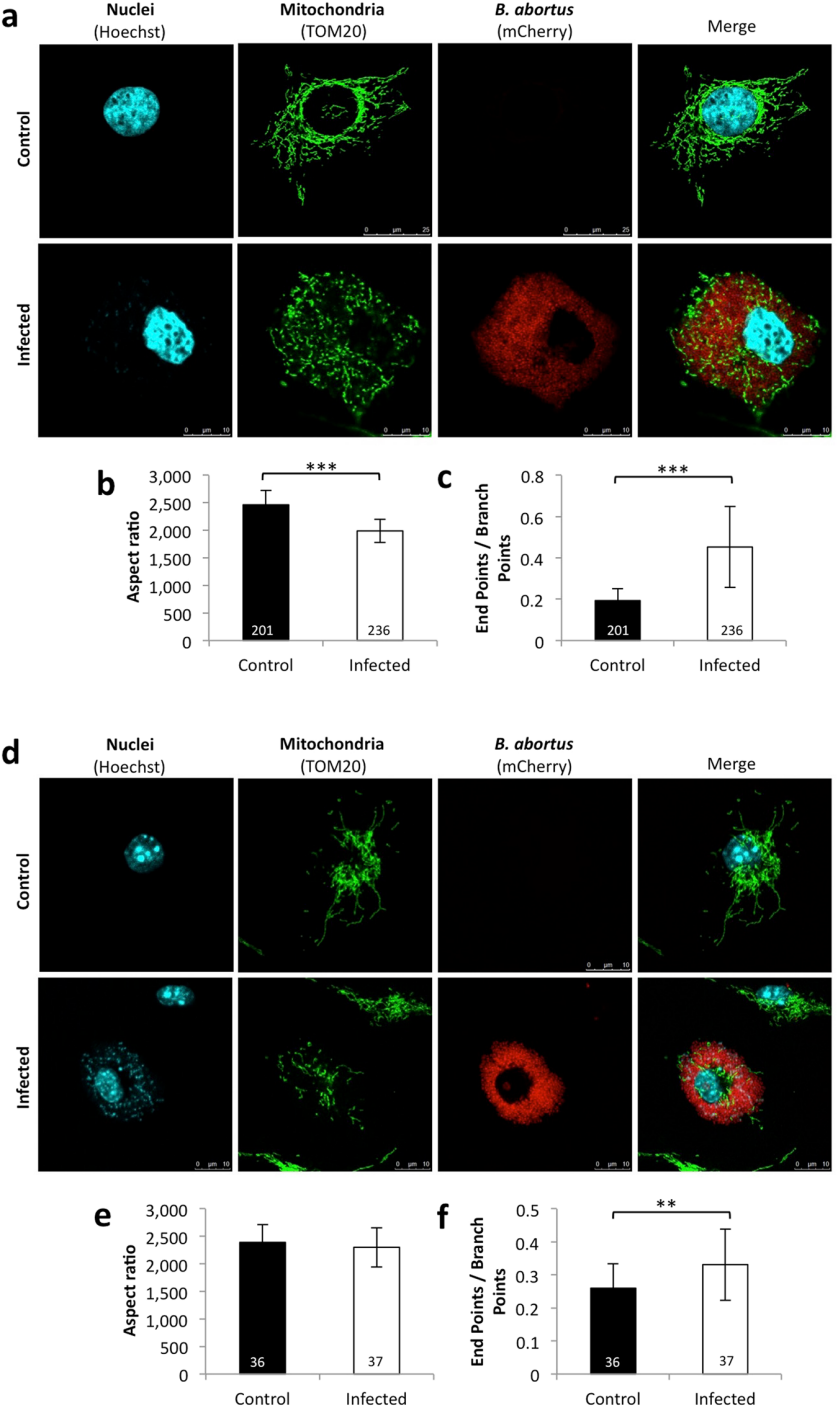
*Brucella abortus* infection induces mitochondrial fragmentation in infected HeLa and BMDM cells (**a**). TOM20 immunostaining in HeLa cells infected or not (control) with *B. abortus* 2308 mCherry - 48 h PI. (Representative of n = 14) Green: TOM20 (Alexa488)/Red: *B. abortus* 2308 (mCherry)/Turquoise: Nuclei (Hoechst) (**b**,**c**). Aspect ratio (**b**) and end point/branch point ratio (**c**) determined in HeLa cells infected or not (control) with *B. abortus* 2308 mCherry - 48 h PI. Results represent means ± SD for fourteen independent experiments (n = 14). Statistical analysis: Rank sum test (Mann-Whitney). (***P < 0.001). The numbers indicated in the columns represent the number of cells analysed for each condition. (**d**) TOM20 immunostaining in BMDM cells infected or not (control) with *B. abortus* 2308 mCherry - 48 h PI (Representative of n = 3). Green: TOM20 (Alexa488)/Red: *B. abortus* 2308 (mCherry)/Turquoise: Nuclei (Hoechst) (**e**,**f**) Aspect ratio (**e**) and end point/branch point ratio (**f**) of BMDM cells infected or not (control) with *B. abortus* 2308 mCherry - 48 h PI. Results represent means ± SD for three independent experiments (n = 3). Statistical analysis: Rank sum test (Mann-Whitney). (**P < 0.01) The numbers indicated in the columns represent the number of cells analysed for each condition.

While the inhibition of the mitochondrial respiration did not affect *Brucella* replication, we wondered whether the intracellular bacteria could affect the mitochondrial network. To do so, we immunostained the infected cells with an antibody targeting the Translocase of the Outer Membrane 20 (TOM20), a protein located in the mitochondrial outer membrane that acts as a receptor for mitochondrial protein import^28^. We first analysed, by confocal microscopy, the mitochondrial morphology in HeLa cells infected with *B. abortus* 2308 mCherry at several PI time points ranging from 2 to 48 h (Supp. Fig. S2a). We then used the ImageJ software to analyse the morphology of the mitochondrial network, determining the “aspect ratio” to estimate the level of the network elongation and the “end point/branch point” ratio to evaluate the level of network connectivity^29^ (Supp. Fig. S2b). This analysis revealed a shortening of the of mitochondrial fragments, detected at 24 h PI and enhanced at 48 h PI, while the fragmentation of the mitochondrial network was clearly detected only at 48 h PI (Supp. Fig. S2c and d). This fragmentation was exclusively observed in cells that contained bacteria. These experiments were repeated (n = 14) in *B. abortus*-infected HeLa cells at 48 h PI, confirming our previous data (Fig. 3a–c).

The same analysis was performed in *B. abortus*-infected bone marrow-derived macrophages (BMDMs) used as *ex vivo* host cells, confirming that mitochondrial fragmentation was not restricted to infected HeLa cells, but was also observed in BMDMs (Fig. 3d–f). However, in the latter, there was no statistically significant difference in the aspect ratio between the control and infected cells. One potential explanation might be that the high nucleus/cytoplasm ratio of the macrophages limits the length of mitochondrial fragments even when the mitochondrial network is connected/elongated.

We further confirmed that the *Brucella*-induced mitochondrial fragmentation also occurred in other cell types, such as RAW 264.7 macrophages (Supp. Fig. S3a) and BeWo trophoblasts (Supp. Fig. S3b), two additional cell models relevant to the study of *Brucella* infection^14^. We also determined whether mitochondrial fragmentation is specific to *B. abortus* by examining the mitochondrial morphology in *Brucella melitensis*-infected HeLa and RAW 264.7 cells (Supp. Fig. S4a–c). We found that *B. melitensis* also induces mitochondrial fragmentation, but to a lesser extent than *B. abortus* (Supp. Fig. S4c). Taken together, these results demonstrate that *Brucella* spp. infection induces mitochondrial fragmentation in various human and murine host cells by 48 h PI.

### Search for possible mechanisms responsible for *Brucella*-induced mitochondrial fragmentation

Given the mitochondrial fragmentation induced by *Brucella* infection, we sought to delineate the mechanisms involved, by studying different cell components known to regulate mitochondrial morphology; namely, the cytoskeleton organisation, mitochondrial calcium uptake and the ER stress response.

#### Cytoskeleton organisation

Mitochondria organisation and distribution in the cell depend on the interactions of the mitochondria with the cytoskeleton^30^. Cytoskeleton elements also actively participate in the mitochondria fission process, as microtubules are involved in the mitochondrial recruitment of DRP1^31^, most likely through the promotion of ER–mitochondria interactions^32^. In addition, microfilaments take part in mitochondrial fission by recruiting DRP1 at the fission site, in collaboration with the ER^33^. Recently, a requirement for F-actin has also been postulated for the DRP1-independent mitochondrial fragmentation that occurs during *Listeria monocytogenes* infection^34^.

Interestingly, the cytoskeleton is known to be affected during *Brucella* infection. Actin polymerisation is involved in *Brucella* uptake in both macrophages and epithelial cells^35–38^. *Brucella* also secretes TIR domain-containing protein (TcpB), an effector that modulates microtubule dynamics by acting as a stabilisation factor, although this activity has only been studied *in vitro*^39^.

That is why we studied the actin and tubulin cytoskeletons in *Brucella*-infected cells by performing co-(immuno)staining of β-actin or α-tubulin and TOM20 in HeLa cells infected with *B. abortus* 2308 mCherry (48 h PI) (Supp. Fig. S5a and b). We did not observe any major modification in the organisation of microfilaments or microtubules in the infected cells when compared to the organisation found in control non-infected cells.

#### Mitochondrial calcium uptake

**Figure 4:**
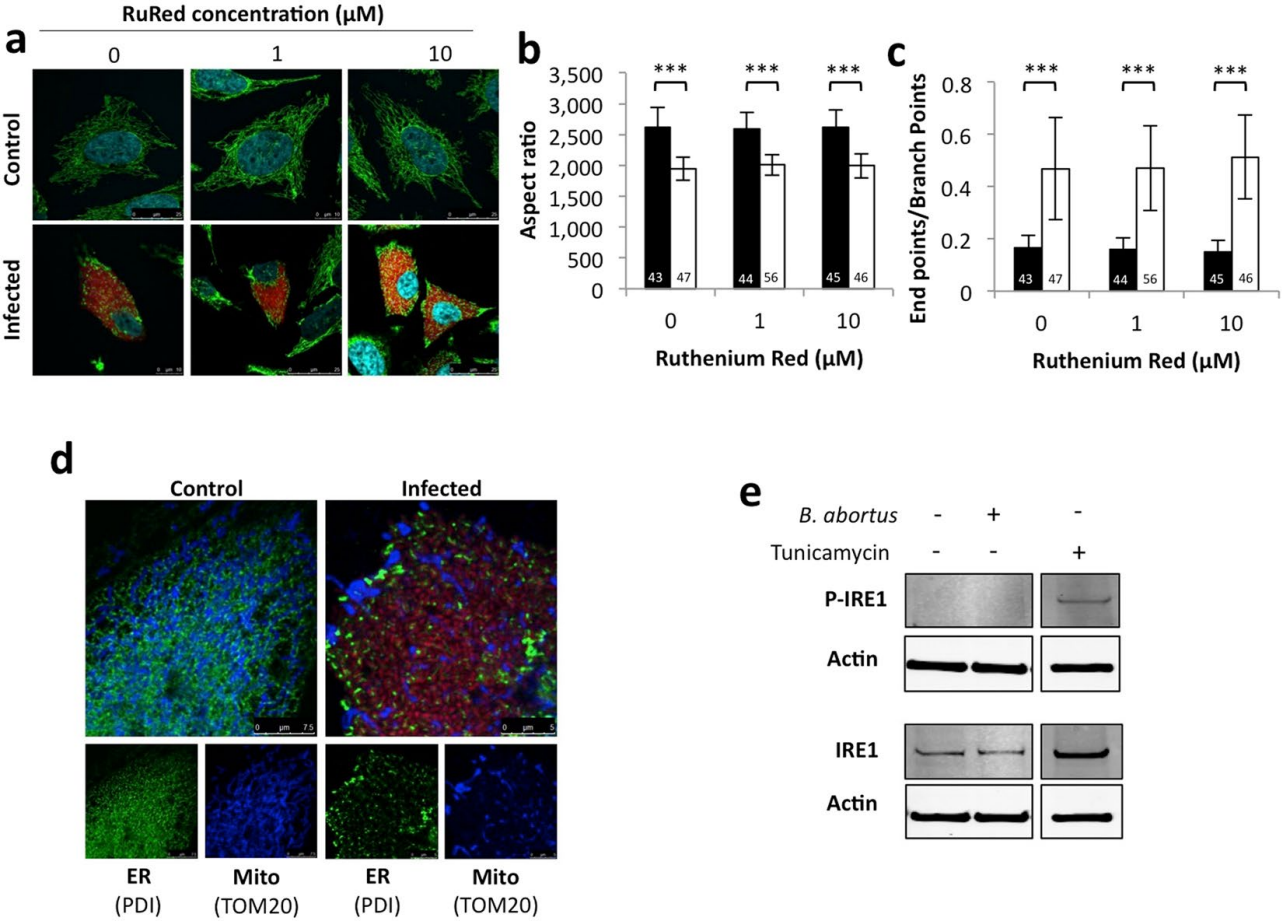
Search for potential mechanisms involved in *Brucella abortus*-induced mitochondrial fragmentation (**a**). TOM20 immunostaining in HeLa cells pre-incubated for 30 min with the indicated concentration of ruthenium red and infected with *B. abortus* 2308 mCherry with or without the inhibitor - 48 h PI (Representative of n = 3). Green: TOM20 (Alexa488)/Red: *B. abortus* 2308 (mCherry)/Turquoise: Nuclei (Hoechst) (**b**,**c**). Aspect ratio (**b**) and end point/branch point ratio (**c**) of HeLa cells pre-incubated for 30 min with the indicated concentration of ruthenium red and infected (white) or not (black) with *B. abortus* 2308 mCherry with or without the inhibitor – 48 h PI. Results represent means ± SD for three independent experiments (n = 3). Statistical analysis: two-way ANOVA on Box Cox transformed data. P value for interaction: 0.446 and 0.051 respectively. The numbers indicated in the columns represent the number of cells analysed for each condition. (**d**) PDI and TOM20 co-immunostaining in HeLa cells infected or not (control) with *B. abortus* 2308 mCherry - 48 h PI (Representative of n = 3). Green: PDI (Alexa488)/Blue: TOM20 (Alexa633)/Red: *B. abortus* 2308 (mCherry)/Turquoise: Nuclei (Hoechst). (**e**) Western blot analysis of P-IRE1 and IRE1 abundance in RAW 264.7 macrophages infected or not with *B. abortus* 2308 mCherry - 48 h PI (Representative of n = 3 in different conditions). RAW 264.7 cells treated for 6 h with 10 µM tunicamycin and then left for 18 h for recovery were used as a positive control. Actin abundance was assessed on the same blot as a loading control. These full-length blots are presented in Supp. Fig. S10a and b.

Elevation of the cytoplasmic calcium concentration ([Ca^2+^]_c_) can lead to mitochondrial fragmentation^40^ in a DRP1-dependent^41^ or, as demonstrated in *L. monocytogenes* infection, in a DRP1-independent manner^34^. Infection with *B. abortus* has been described to induce an increase in [Ca^2+^]_c_ in infected macrophages^42^. Therefore, we analysed the effect of inhibiting the mitochondrial calcium influx on *Brucella*-induced mitochondrial fragmentation. HeLa cells were pre-incubated for 30 min with or without 1 or 10 µM ruthenium red (RuRed) to inhibit the mitochondrial calcium uniporter (MCU)^43–45^. The cells were then infected or not with *B. abortus* 2308 mCherry in the presence or in the absence of the inhibitor. Mitochondrial morphology was assessed at 48 h PI as already described (Fig. 4a–c). Under these conditions, we did not observe any effect of RuRed on *Brucella*-induced mitochondrial fragmentation, suggesting that mitochondrial calcium uptake might not be responsible for the mitochondrial morphology alteration observed during *Brucella* infection.

#### ER stress

The replication of *Brucella* has been shown to lead to the activation of one or several UPR pathways in infected macrophages and epithelial cells^15–17^. As shown by the co-immunostaining of PDI (an ER marker) and TOM20 (a mitochondrial marker), we also observed alterations of the ER distribution in *B. abortus*-infected HeLa cells at 48 h PI (Fig. 4d). ER stress is known to affect mitochondrial morphology and functions^18,19^, so we studied the potential involvement of the UPR on *Brucella*-induced mitochondrial fragmentation.

We characterised UPR activation in our infection model by assessing the phosphorylation status of IRE1. RAW 264.7 macrophages were infected with *B. abortus* 2308 mCherry and at 48 h PI the abundance of phosphorylated and total forms of IRE1 was assessed by western blotting. Cells treated with tunicamycin, an inhibitor of N-glycosylation, were used, as a positive control^16^. As shown in Fig. 4e, we observed no phosphorylation of IRE in response to *Brucella* infection. These results were confirmed in HeLa cells (Supp. Fig. S6a) and were consistent with the absence of activation of other UPR markers, such as the increase in the abundance of the binding immunoglobulin protein (Bip/GRP78) and the phosphorylated form of the eukaryotic translation initiation factor 2 alpha (P-eIF2α) (unpublished data). These results suggest that the UPR is not induced in our experimental model of *B. abortus* infection. However, only a small proportion of cells contain replicating bacteria (around 5 % at 48 h PI for RAW 264.7 macrophages); therefore, we cannot rule out that if phosphorylation occurs only in cells containing the bacteria, the signal for phosphorylated IRE1 might be difficult to detect using a global test like western blotting.

### *Brucella*-induced mitochondrial fragmentation is DRP1-independent

We further explored the mechanisms by which *Brucella* could induce mitochondrial fragmentation by examining the protein abundance of some key factors that regulate and control mitochondrial morphology/dynamics in *B. abortus*-infected cells.

**Figure 5:**
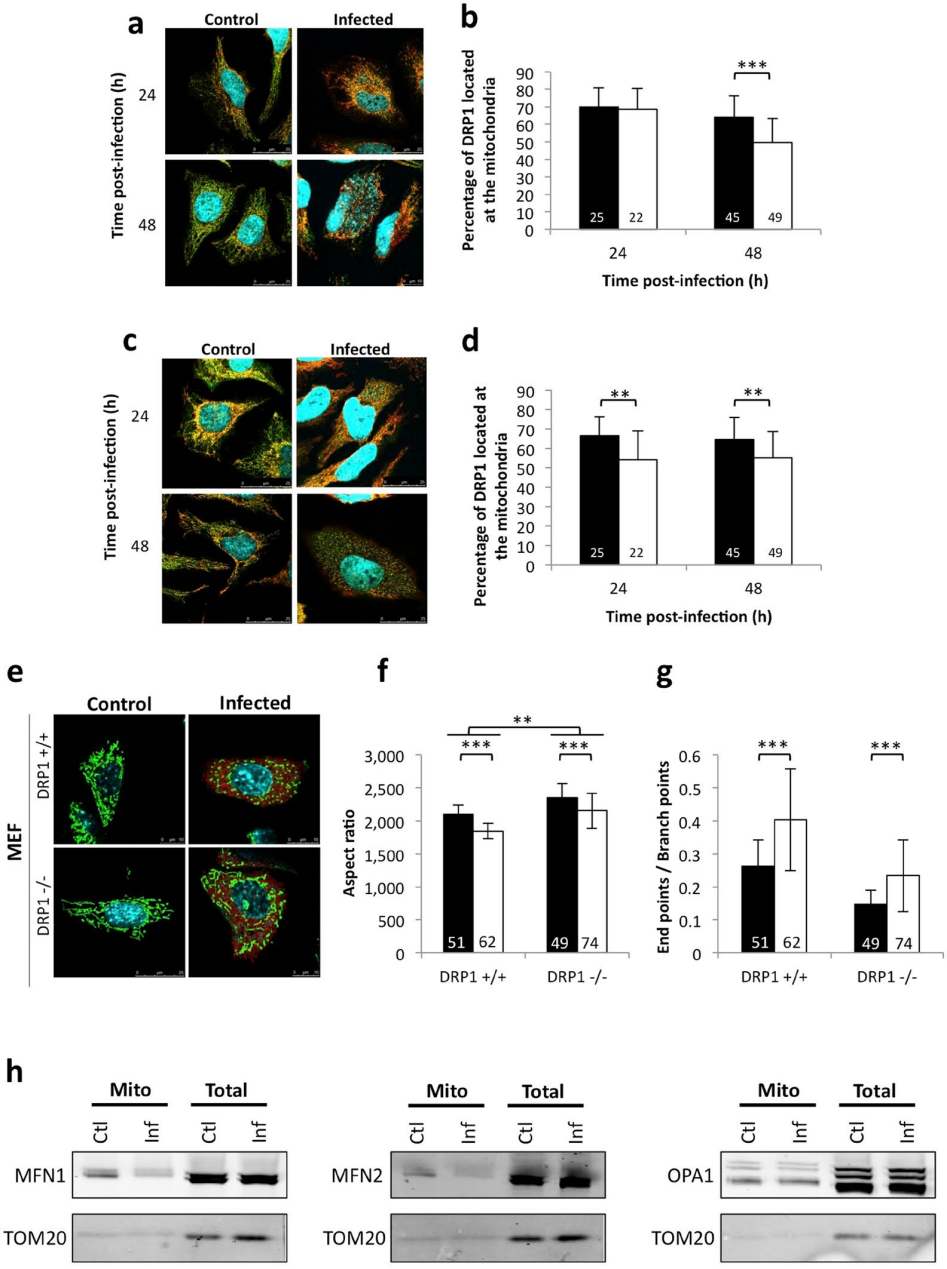
Molecular actors involved in *Brucella abortus*-induced mitochondrial fragmentation (**a**,**c**) FIS1 (**a**)/MFF (**b**) and DRP1 co-immunostaining in HeLa cells infected or not (control) with *B. abortus* 2308–24 and 48 h PI (Representative of n = 3). Green: DRP1 (Alexa488)/Red: FIS1/MFF (Alexa563)/Turquoise: Nuclei and *B. abortus* 2308 (Hoechst) (**b**,**d**) Quantification of the percentages of DRP1 co-localising with FIS1 (**b**)/MFF (**d**) in HeLa cells infected (white) or not (black) with *B. abortus* 2308. Results represent means ± SD for three independent experiments (n = 3). Statistical analysis: Rank sum test (Mann-Whitney) (**P < 0.01, ***P < 0.001). The numbers indicated in the columns represent the number of cells analysed for each condition (**e**). TOM20 immunostaining in DRP1^+/+^ or DRP1^−/−^ MEFs infected or not (control) with *B. abortus* 2308–48 h PI (Representative of n = 3). Green: TOM20 (Alexa488)/Red: *B. abortus* 2308 (mCherry)/Turquoise: Nuclei (Hoechst) (**f**,**g**) Aspect ratio (**f**) and end point/branch point ratio (**g**) of DRP1^+/+^ or DRP1^−/−^ MEFs infected (white) or not (black) with *B. abortus* 2308 mCherry - 48 h PI. Results represent means ± SD for three independent experiments (n = 3). Statistical analysis: two-way ANOVA on Box Cox transformed data. P value for interaction: 0.004 and 0.692 respectively. The numbers indicated in the columns represent the number of cells analysed for each condition. (**h**) Western blot analysis of MFN1, MFN2 and OPA1 abundance in mitochondrial enriched fractions (Mito) or total proteins from total cell lysates of RAW 264.7 cells infected (Inf) or not (Ctl) with *B. abortus* 2308 mCherry - 48 h PI. TOM20 abundance was assessed on the same blot as the loading control. These full-length blots are presented in Supp. Fig. S10c,e.

We first compared the percentages of DRP1, a key effector of mitochondrial fission^3^, located at the mitochondria in cells with or without *Brucella* infection. HeLa cells were infected with *B. abortus* 2308 WT, followed by co-immunostaining for DRP1 and MFF or FIS1 (two known DRP1 receptors)^3^ and acquisition of confocal micrographs at 24 and 48 h PI. The percentages of DRP1 that co-localised with the above-mentioned receptors were quantified using the ImageJ software (Figs 5a–d and S7 and S8). A decrease in the proportion of DRP1 that co-localised with the mitochondria in infected cells, when compared to the control, suggests that DRP1 is not recruited to the mitochondria in *Brucella*-infected cells and therefore might not be involved in the fragmentation of the organelle triggered by the bacteria.

We confirmed this hypothesis by analysing the capacity of *Brucella* to induce mitochondrial fragmentation in the absence of DRP1. DRP1^+/+^ and DRP1^−/−^ mouse embryonic fibroblasts (MEFs) were infected with *B. abortus* 2308 mCherry. The distribution of the mitochondrial network immunostained for TOM20 was observed by confocal microscopy at 48 h PI (Fig. 5e–g). As expected, the mitochondrial network was more elongated for DRP1^−/−^ cells than for DRP1^+/+^ cells (p value < 0.001). However, *Brucella* induced a fragmentation of the organelle in both cell lines, confirming that DRP1 is not required for *Brucella*-induced mitochondrial fragmentation. Nevertheless, the absence of DRP1 has a significant effect on modifications of the network morphology induced by the bacteria (aspect ratio - P value for interaction: 0.004) (Fig. 5f), so we cannot completely rule out a partial participation of DRP1 in the fragmentation process.

We next analysed the mitochondrial abundance of the fusion effectors in *Brucella*-infected cells. RAW 264.7 macrophages were infected with *B. abortus* 2308 mCherry. At 48 h PI, mitochondrion-enriched fractions were prepared and the abundance of MFN1, MFN2 and OPA1 was analysed by western blotting. TOM20 abundance was used as a loading control (Fig. 5 h). The abundance of MFN1 and MFN2, but not OPA1, is dramatically and exclusively reduced in the mitochondrial fractions of *Brucella* infected cells, suggesting a change in the location of the protein during *Brucella* infection rather than a reduction of their global expression. These results suggest that the fragmentation of mitochondria induced by *Brucella* might result from a deficit of mitochondrial fusion, rather than an increase in mitochondrial fission.

### Mitochondrial fragmentation does not affect *Brucella* replication

**Figure 6:**
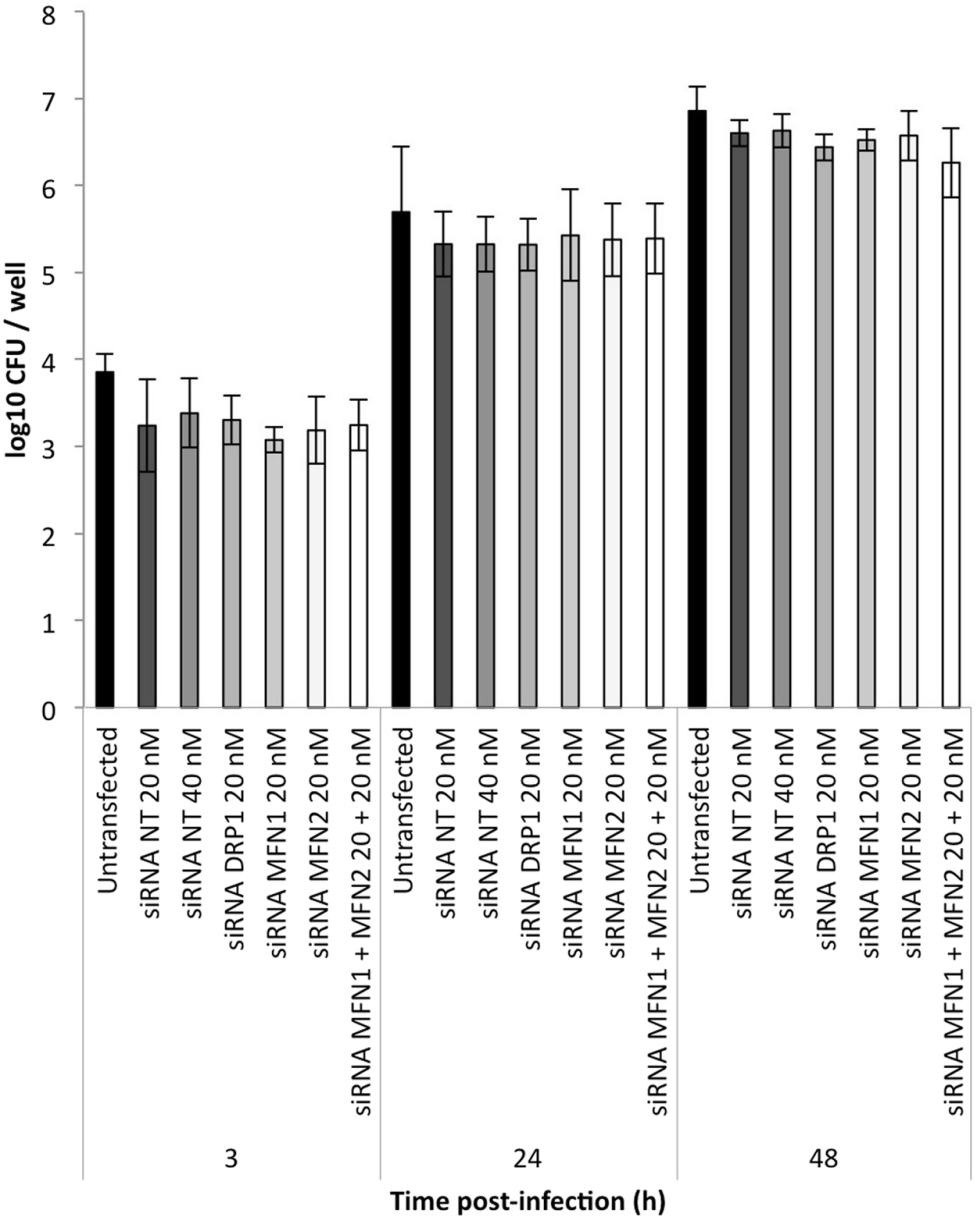
Alteration of mitochondrial morphology does not affect *Brucella abortus* replication. CFU/well of Hela cells transfected or not (Untransfected) with siRNA non-target (20 or 40 nM) or siRNAs against DRP1 (20 nM), MFN1 (20 nM), MFN2 (20 nM) or MFN1/2 (20 nM + 20 nM) and infected with *B. abortus* 2308. Results represent means ± SD for three independent experiments (n = 3). Statistical analysis: two-way ANOVA.

We next wondered whether the fragmentation of mitochondria in the host cells was part of a cell defence mechanism or part of a bacterial invasion strategy. We analysed the effect of the mitochondrial fragmentation on *Brucella* replication by inducing modifications of the mitochondrial morphology using siRNA against different effectors that regulate mitochondrial dynamics. As expected, the silencing of DRP1 in HeLa cells led to an elongation of the mitochondrial network, whereas siRNAs directed against MFN1, MFN2 or both resulted in a fragmented network (Supp. Fig. S9). HeLa cells were then transfected with specific siRNAs against mRNAs encoding these proteins or non-target siRNA and infected with *B. abortus* 2308. *Brucella* replication was assessed under the different conditions by counting the CFU at 3, 24 or 48 h PI, but we found no significant differences between the CFU obtained for the different conditions affecting the mitochondrial morphology and the CFU for cells transfected with non-target siRNA (Fig. 6). A slight difference was noted between the CFU recovered from transfected and untransfected cells, most likely due the stress caused by transfection. These results demonstrate that the entry and replication of *B. abortus* are not affected by changes in mitochondrial morphology at the onset of the infection.

### Mitochondrial fragmentation does not protect *Brucella*-infected macrophages against TNFα-induced apoptosis

The morphological status of mitochondria is known to modulate apoptosis either by promoting apoptosis, as reviewed by Scorrano^46^, or by protecting against apoptosis, as demonstrated by Szabadkai^6^. In addition, *Brucella* may impact host cell apoptosis by inhibiting^47–54^ or inducing^55–59^ programmed cell death according to the cell type. One possibility is that mitochondrial fragmentation triggered by the bacteria might modulate the capacity of infected cells to respond to the extrinsic stresses encountered during the infection, such as exposure to pro-inflammatory cytokines. We examined the relative sensitivity of cells containing the bacteria to TNFα, an apoptosis inducer and pro-inflammatory cytokine, secreted by some *Brucella*-infected cells^59–61^.

**Figure 7:**
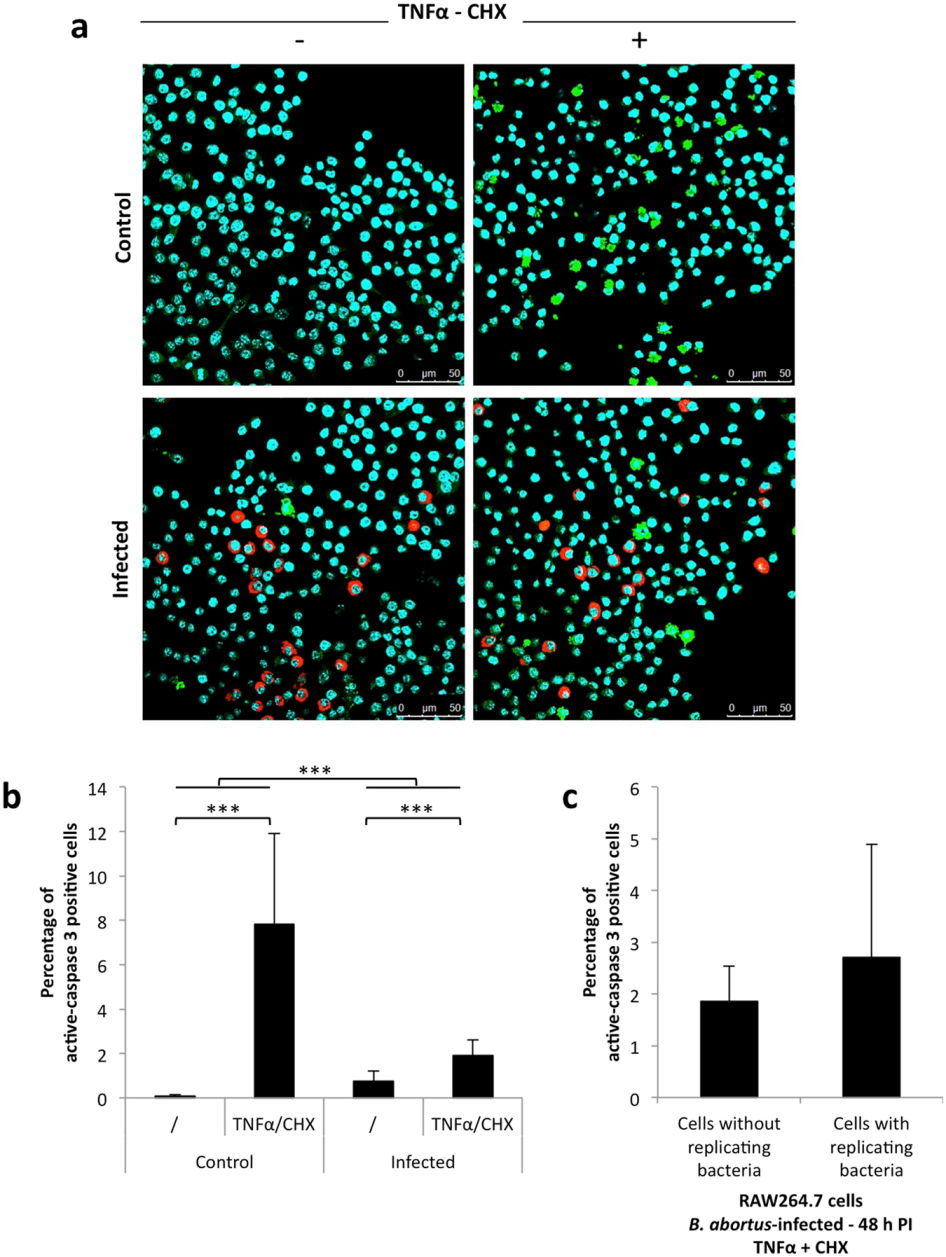
Effect of mitochondrial morphology alteration on susceptibility of *Brucella abortus-*infected cells to apoptosis (**a**). Active caspase 3 immunostaining in RAW 264.7 macrophages infected or not (control) with *B. abortus* 2308 mCherry and stimulated with or without 10 ng/ml TNFα and 10 µg/ml CHX for 2 h (Representative of n = 4). Green: Active Caspase 3 (Alexa488)/Red: *B. abortus* 2308 (mCherry)/Turquoise: Nuclei (Hoechst) (**b**). Quantitative analysis of the percentages of cells positive for active caspase 3 staining in RAW 264.7 macrophages infected or not (control) with *B. abortus* 2308 mCherry and stimulated with or without 10 ng/ml TNFα and 10 µg/ml CHX for 2 h. Results represent means ± SD for four independent experiments (n = 4). Statistical analysis: two-way ANOVA on log transformed data. (***P < 0.001) (**c**). Quantitative analysis of the percentages of cells expressing the active caspase 3 in RAW 264.7 macrophages infected with *B. abortus* 2308 mCherry for 48 h and stimulated with 10 ng/ml TNFα and 10 µg/ml CHX for 2 h, among cells containing or not replicating bacteria. Results represent means ± SD for four independent experiments (n = 4). Statistical analysis: Student’s t-test.

RAW 264.7 macrophages were infected with *B. abortus* 2308 mCherry and at 48 h PI (when the mitochondrial network is highly fragmented), the cells were incubated for 2 h with or without 10 ng/ml TNFα and 10 µg/ml cycloheximide (CHX) to induce apoptosis. CHX is an inhibitor of protein synthesis reported to inhibit the pro-survival NF-κB pathway that is activated by TNFα^62^. Apoptosis was assessed by confocal microscopy analysis of active caspase 3 (A-C3) immunostaining (Fig. 7a). We first assessed the percentages of total A-C3-positive cells under the different conditions and showed that *Brucella* seems to protect cells against TNFα-induced apoptosis. *Brucella* infection decreased the percentage of A-C3 positive cells induced by a TNFα/CHX treatment from 7.82 % in uninfected cells to 1.91 % in the infected cells (Fig. 7b). However, when considering only the cell population exposed to the bacteria (containing or not bacteria) and treated with TNFα/CHX, we observed no difference in the percentages of A-C3 positive cells between cells that contained replicating bacteria (2.71 %) and cells that did not contain replicating bacteria (1.87 %) (Fig. 7c). These results suggest that it is not the mitochondrial fragmentation triggered by the bacteria that explains the protective effect of *Brucella* in the TNFα-treated cells.

## Discussion

This study of the crosstalk between *Brucella* and mitochondria in infected cells highlighted potential physical interactions between mitochondria and BCVs. We confirmed that *Brucella* does not rely on mitochondrial OXPHOS for its intracellular replication. We also showed that mtROS do not participate to the control of *Brucella* replication *in vitro*. Finally, we demonstrated that *Brucella* induces a DRP1-independent mitochondrial fragmentation in infected cells and that this fragmentation does neither affect the bacterial replication nor the susceptibility of infected cells to TNFα-induced apoptosis.

First, electron microscopy examinations revealed the presence of very intimate contacts between mitochondria and BCVs, suggesting a possible physical interaction between these two structures. Further analyses are needed to characterise the molecular nature of these putative interactions, but these results corroborate previous observations^23^. A previous attempt to characterise the protein content of BCVs in BHK21 cells by a proteomic approach was hampered by contamination of the BCV fraction with mitochondria during the cell fractionation procedure. Furthermore, attempts to eliminate the mitochondria from the BCV fractions by immunoprecipitation of the organelle using Dynabeads coated with antibodies raised against VDAC1 led to a loss of most of the BCVs^23^, a finding that supports the hypothesis of a physical interaction between mitochondria and BCVs in the infected cells.

We showed that inhibition of the respiratory chain does not change the efficiency of *Brucella* infection and replication. One explanation for this result may involve the observation that we (unpublished) and others have made whereby *B. abortus* infection leads to the activation of glycolysis in infected macrophages^63^. This activation is linked to M2 polarisation of infected macrophages, which are known to rely on glycolysis for their metabolism^63^. Those results also corroborate the results obtained by Czyz and collaborators that demonstrate that *B. abortus* induces a Warburg-like metabolic shift in human macrophages. This shift promotes the bacterial survival, probably by providing amino acids and lactate that can be metabolised by the bacteria^20^. This process is not specific to *Brucella* as it is also described during *Mycobacterium tuberculosis* infection^64^.

We also highlighted that the increase in production of mtROS that results from the inhibition of mitochondrial electron transport chain in *B. abortus*-infected macrophages does not seem to control the intracellular replication of the bacteria. However, one interesting feature is that mtROS participate in the establishment of the immune response *in vivo*^22^. Indeed, infection with *B. abortus* triggers the production of mtROS in BMDM after 17 h and contributes to the secretion of IL-1β in an NLRP3-dependent manner. NLRP3 or IL-1β KO mice are reportedly more susceptible to *B. abortus* infection^22^. Unfortunately, the use of the MitoSOX probe, which is typically used for the specific detection of O_2_^−^, in the mitochondria, is restricted to unfixed cells and we were unable to measure mtROS production directly under our experimental conditions.

We demonstrated that *B. abortus* and *B. melitensis* replication is accompanied by mitochondrial fragmentation at 48 h PI in several infected human and murine cell types. Although alterations of the mitochondrial distribution during *Brucella* infection were recently described, the mechanisms involved in the process were not analysed^20,65^. We thus explored several hypotheses to identify the potential mechanisms underlying the observed *Brucella*-induced mitochondrial network fragmentation.

First, we showed that *Brucella* infection does not modify the actin and tubulin cytoskeleton organisation at 48 h PI. Thus, changes in the organisation of these structures are unlikely to be connected to mitochondrial fragmentation. Additionally, the inhibition of actin or tubulin polymerization at the end of the infection (the only time compatible with the use of pharmacologically active concentrations that maintain cell viability) using cytochalasin D (24 h–400 ng/ml) or nocodazole (4 h–1 µg/ml), respectively, does not prevent *Brucella*-induced mitochondrial fragmentation (data not shown). Together, those results suggest that actin and tubulin cytoskeleton components do not take part in the modification of mitochondrial morphology observed in *Brucella*-infected cells. However, we cannot completely rule out that conditions allowing to prevent the actin/tubulin polymerization during the entire post-infection time would not have affected *Brucella*-induced mitochondrial fragmentation.

We also showed that MCU inhibition does not prevent mitochondrial fragmentation in *Brucella-*infected cells, which suggests that mitochondrial calcium uptake might not be responsible for this process. However, even if mitochondrial calcium influx is mainly mediated by MCU, other accessory channels and transporters might also participate in the process^66^. Additionally, cytosolic calcium-dependent signalling could still play a part in *Brucella*-induced mitochondrial fragmentation by a mechanism independent of mitochondrial calcium buffering. For example, high [Ca^2+^]_c_ levels can activate signalling pathways that modulate the activity of effectors of mitochondrial morphology, such as the mitogen-activated protein kinase (MAPK) extracellular signal-regulated kinase 1/2 (ERK1/2) that can phosphorylate DRP1 and promote its recruitment to the mitochondrial surface in cells exposed to high glucose concentrations^67^. Therefore, we cannot completely rule out a possible role for changes in calcium homeostasis in *Brucella*-induced mitochondrial fragmentation.

The role of UPR also seems marginal in terms of the fragmentation of mitochondria observed in infected cells, as it was not activated under the infection conditions used in this study. We did not detect any phosphorylation of IRE1 in infected RAW 264.7 or HeLa cells, a result consistent with the absence of any activation of UPR markers in *B. abortus*-infected RAW 264.7 cells (unpublished data). However, alterations of the ER structure caused by the massive proliferation of *B. abortus* within the organelle could indirectly induce mitochondrial fragmentation by disturbing the physical contacts between ER and mitochondria.

*Brucella*-induced mitochondrial fragmentation is DRP1-independent and might be caused by a deficit of mitochondrial fusion. Calcium flux-dependent and DRP1-independent mitochondrial fragmentation was previously described in the context of *L. monocytogenes* following insertion of lysteriolysin O (LLO), a pore-forming toxin, in the plasma membrane, which triggered a transient mitochondrial fragmentation during bacterial entry^34,68^. Mitochondrial fragmentation caused by a fusion deficiency was also reported in several models, including Alzheimer disease^69^, embryonic nicotine exposure^70^, extrahepatic cholestasis^71^ and neuroblastoma^72^, in which depletion of MFN1 and/or MFN2 was sufficient to trigger fragmentation of the mitochondrial network.

The potential involvement of bacterial components and/or effectors in active manipulation of mitochondrial morphology still needs more investigation. The poor characterisation of *Brucella* effectors^73^ and the fact that VirB is essential for *Brucella* replication^14^ complicate the elucidation of molecular mechanisms by which *Brucella* could induce mitochondrial fragmentation. However, a recently published conditional *virB11* mutant could be very useful for studying the involvement of bacterial effector release in post-replication processes, such as mitochondrial fragmentation^74^.

Although our current set of data does not allow to exclude that *Brucella*-induced mitochondrial fragmentation is part of a global stress response caused by the bacteria engulfment or heavy bacterial load in the cytoplasm of infected cells, several reports in the literature suggest that it is unlikely that the bacterial entry, by itself, induces mitochondrial fragmentation. Indeed, in their study about *L. monocytogenes*-induced mitochondrial fragmentation in HeLa cells, Stavru and collaborators showed that this process is not a general response to bacterial entry as different bacterial species including extracellular bacteria (EPEC), intracellular bacteria evolving in a vacuole (*E. coli*(Inv) (model for *Yersina pseudotuberculosis*) and *Salmonella*) or in the cytoplasm (*S. flexneri* and *L. innocua*) do not induce mitochondrial fragmentation^68^. Additionally, there are examples of bacterial effectors inducing mitochondrial fragmentation such as LLO from *L. monocytogenes*^68^, VacA from *H. pylori*^75^ and MitF from *L. pneumophila*^76^.

Furthermore, it is important to emphasize that even if the mitochondrial fragmentation observed in *Brucella*-infected cells is caused by the stress of a massive infection, the phenotype is still physiological and thus interesting to study in details. Indeed, it is known that *Brucella* is a stealthy bacterium that can replicate massively inside the host cell without impacting its viability. In addition, such highly infected cells can be observed *in vivo* in different infection models such as alveolar macrophages of intranasally-infected mice^77^ or trophoblasts of infected pregnant mice^78^ or goats^79^.

Mitochondrial morphology might not control *Brucella* replication. Notably, even when the mitochondrial morphology is modified at the time of the infection, *Brucella* is still able to trigger the mitochondrial fragmentation during the course of the infection, as observed in DRP1^−/−^ MEF cells, therefore limiting the analysis of the putative impact of altered mitochondrial morphology on bacterial replication. We found that the mitochondrial fragmentation observed in cells containing *Brucella* neither protects against nor amplifies TNFα-induced apoptosis, as monitored by caspase-3 activation. Indeed, even when *Brucella* infection seems to protect against apoptosis, this protection is observed across the entire cell population and not specifically in cells containing the bacteria. This protection is therefore probably mediated by soluble factors secreted by the bacteria or by the infected cells. Indeed, *Brucella* is known to prevent the activation of IFNγ or FasL-induced apoptosis both in infected and non-infected monocytes, suggesting the involvement of soluble factors^49^. Additionally, *Brucella-*infected cells are known to secrete TNFα^50,56,58,59,80–82^, a cytokine with a known dual effect. On the one hand, TNFα induces pro-survival NF-κB signalling and, on the other hand, in cases of excessive stimulation, it promotes apoptosis^83^. The hormesis effect predicts that exposure to an unlethal stress will enhance the capacity of a cell to deal with a more severe one^84^. Therefore, TNFα secreted by infected cells might improve cellular resistance to TNFα-induced apoptosis by promoting pro-survival pathways.

The benefit that the infected cell or *Brucella* obtains from this alteration in the mitochondrial morphology during the replication of the bacteria is unclear. In the context of infection, other bacteria, such as *Helicobacter pylori*^75^, *L. monocytogenes*^68^ or *Vibrio cholerae*^85^, are reported to modulate mitochondrial morphology in order to induce apoptosis, generate a metabolic slowdown of the host cell or interfere with the immune signalling, respectively. From a more global point of view, the importance of mitochondrial morphology has been illustrated in different human pathologies, such as Parkinson disease^86^, Alzheimer disease^87^ or diabetes^88^. The impact of mitochondrial dynamics on cell metabolism and mtROS production is the most documented^89–92^, but changes in the morphology of mitochondria now appear to modulate a variety of other cell functions by affecting signalling pathways, such as the MAVS or Nuclear factor erythroid-2-related factor 2 (NRF2)^93^ pathways, thereby affecting phenotype in various ways, including the cell inflammatory response or the differentiation status.

In conclusion, *Brucella* replication modifies the mitochondrial morphology of infected cells. More research efforts are now needed to fully characterise the mechanisms involved in this process and the consequences for either the host cells or the pathogen. The present study highlights a new aspect of the host-pathogen relationship occurring during *Brucella* infection.

## Methods

### Ethics statement

The procedures used in this study and the handling of the mice complied with current European legislation (directive 86/609/EEC) and the corresponding Belgian law “Arrêté royal relatif à la protection des animaux d’expérience du 6 Avril 2010 publié le 14 Mai 2010.” The Animal Welfare Committee of the University of Namur (Namur, Belgium) reviewed and approved the complete protocol (Permit Numbers 13/199 and 16/277).

### Cell culture

RAW 264.7 macrophages (ATCC, Manassas, VA, USA) were cultured in Dulbecco’s Modified Eagle Medium High Glucose (4.5 g/L) and NaHCO_3_ (1.5 g/L) (DHG-L1, Gibco-Life Technologies, Carlsbad, CA, USA), supplemented with 10 % heat-inactivated foetal bovine serum (FBS, Gibco). HeLa cells (ATCC) were cultured in Minimum Essential Medium (MEM, Gibco) supplemented with nonessential amino acids (Gibco), 1 mM pyruvate (Gibco) and 10% FBS. DRP1^+/+^ and DRP1^−/−^ MEFs (a generous gift from Prof. Ishihara, Kurume University, Japan) were kept in Dulbecco’s Modified Eagle Medium (DMEM, Gibco) supplemented with 10 % FBS and EB1 and rho^0^ HeLa cells (kind gift from Prof. Hayashi, University of Tsukuba, Japan) in DMEM high glucose (DHG, Gibco) supplemented with 1 mM pyruvate, 50 µg/ml uridine (Sigma-Aldrich, St. Louis, MO, USA) and 10% FBS. BMDMs were obtained from femurs and tibias of 6-to-8-week-old C57BL/6 mice, as previously described^94^. Cells were seeded and treated in culture plates (Corning-Costar, Lowell, MA, USA).

When indicated, myxothiazol (10 nM, Sigma-Aldrich), antimycin A (100 nM, Sigma-Aldrich), Mito-TEMPO (500 µM, Sigma-Aldrich), ruthenium red (1 or 10 µM, Sigma-Aldrich), tunicamycin (10 µM, Sigma-Aldrich), TNFα (10 ng/ml, Sigma-Aldrich) or cycloheximide (10 µg/ml, Sigma-Aldrich) were added to the culture media for the indicated times.

### Bacterial strains

*Brucella abortus* 2308 is a CO_2_-independent, virulent, smooth strain. The *B. abortus* 2308 mCherry strain constitutively expresses fluorescent mCherry due to the integration of a plasmid containing the mCherry coding sequence and a kanamycin resistance marker^95^. Cultures of *Brucella* were freshly inoculated from frozen stock into yeast extract and tryptone (2YT) medium [1 % yeast extract (Invitrogen, Carlsbad, CA, USA), 1.6 % bactotryptone (Invitrogen), 0.5 % NaCl (Invitrogen)) plates (supplemented with 10 µg/ml kanamycin (AppliChem Panreac) for the mCherry strain], before subcultures were grown in 2YT broth (aerobic condition, 37 °C).

All *Brucella* were handled under BSL-3 containment according to the Council Directive 98/81*/*EC of 26 October 1998, adopted by the Walloon Government (4 July 2002).

### Cell infection

Bacterial growth was measured by monitoring the culture optical density at 600 nm. Bacterial cultures were pelleted and resuspended in phosphate-buffered saline (PBS) before adjusting the bacterial suspension to the appropriate multiplicity of infection (MOI) (HeLa: 1000, RAW 264.7: 300, BMDM: 300 and MEF: 300) in the corresponding medium supplemented with 10 % FBS. The infectious dose was monitored by plating bacteria on 2YT plates and then counting CFUs. For host cell infections, bacteria were sedimented onto cells through a centrifugation at 400 g for 10 min at 4 °C to favour the cell-bacteria contacts and then incubated for 1 h at 37 °C in 5 % CO_2_. Thereafter, cells were washed twice with PBS and incubated for the different times PI with 50 µg/ml gentamycin (Invitrogen) to eliminate the remaining extracellular bacteria.

### CFU counts

*Brucella* replication was assessed by CFU counting at several PI time points. Cells were washed three times with PBS and lysed for 10 min with PBS containing 0.1 % Triton X-100 (Sigma-Aldrich). Viable bacteria were quantified by plating serial dilutions of this lysate on 2YT plates.

### Transmission Electron Microscopy

#### Cell infection

HeLa cells were seeded in 6-well plates on 35 mm coverslips (Ibidi) (150,000 cells/well). On the next morning, cells were infected with *B. abortus*-RFP for 24 h. The cells were fixed overnight in 2.5 % glutaraldehyde in cacodylate buffer (150 mM sodium cacodylate, 2 mM MgCl_2_) at 4 °C.

#### Mouse infection and organ collection

Bacterial growth was measured through the culture optical density at 600 nm. Bacterial cultures were pelleted, washed in RPMI and resuspended for injection in the same medium at a density of 2 × 10^5^ bacteria/ml. The infectious dose was checked by plating bacteria on 2YT plates and by counting CFUs.

Oestrus of 8–14 weeks old BALB/c females was synchronised 3 days before mating and pairs were set up with 3- to 4-month-old males. The following morning, the presence of a vaginal plug was checked and the potentially fertilised females were isolated. That day corresponded to day 0 post-fecundation (PF). At day 10 PF, pregnant females were infected intraperitoneally with 500 µl of bacterial suspension (10^5^ bacteria). At day 15 PF, mice were anaesthetised with isoflurane and sacrificed by cervical dislocation, as previously described in Barbier *et al*.^78^. All infections were performed at an Animal Biosafety Level 3 facility. Conceptuses were removed from maternal uterine horns and incubated overnight at 4 °C in PBS supplemented with 2.5% glutaraldehyde for fixation. Tissue fragments were washed several times in cacodylate buffer (150 mM sodium cacodylate, 2 mM MgCl_2_) at 4 °C. The samples were then fixed overnight in 2.5 % glutaraldehyde in cacodylate buffer (150 mM sodium cacodylate, 2 mM MgCl_2_) at 4 °C.

#### Sample preparation

Following overnight fixation, samples were washed three times with cacodylate buffer (150 mM sodium cacodylate, 2 mM MgCl_2_) at 4 °C. The samples were then immersed in freshly prepared reduced osmium buffer (2 % osmium tetroxide, 40 mM potassium ferrocyanide, 150 mM sodium cacodylate, 2 mM MgCl_2_) for 1 h at 4 °C. The samples were subsequently washed three times with water at room temperature and immersed in 100 mM thiocarbohydrazide for 20 min. After three washes with deionised water, the samples were post fixed in 2 % osmium tetroxide for 30 min. at room temperature. The samples were then washed with water and incubated in 1 % uranyl acetate overnight at 4 °C. After three washes with water, the samples were incubated in freshly prepared 20 mM lead aspartate solution for 30 min. at 60 °C. The samples were then washed three times, dehydrated with ethanol and immersed in 50% durcupan:ethanol solution for 1 hour. The samples were then embedded in 100 % durcupan. The resin was polymerised at 60 °C for 48 h.

#### Electron microscopy

Resin-embedded samples were trimmed and mounted to pre-tilt 45° SEM stubs (Agar Scientific AGG3020) using colloidal silver paint. Electron microscopy images were acquired using a Helios NanoLab DualBeam instrument (FEI). Regions of interest were polished using the focused ion beam (FIB) operating at 0.79 nA. Images (3072 by 2048 pixels) were collected using an Elstar in-lens BSE detector at 1.5 kV with a horizontal field width of 15 μm at a working distance of 4.01 mm.

### Immunostaining

Cells were seeded on glass coverslips in 12-well plates 24 h before *Brucella* infection (HeLa: 30,000 cells/well, BMDM: 100,000 cells/well, MEFs: 10,000 cells/well and RAW 264.7: 50,000 cells/well). At several times PI, cells were fixed with 4 % paraformaldehyde for 20 min, permeabilised for 5 min with PBS-1 % Triton X-100, and then incubated for 2 h at RT with the appropriate primary antibody (16 h at 4 °C for the active caspase-3 antibody) diluted in PBS-2 % BSA. Cells were then incubated for 1 h at RT with an Alexa-labelled secondary antibody diluted in PBS-2 % BSA. Cells were then incubated for 30 min with Hoechst FluoroPure grade (Invitrogen Molecular Probes) and phalloidin (Invitrogen Molecular Probes) diluted 1:5,000 and 1:50, respectively, in PBS. Finally, the cells on coverslips were mounted in Mowiol (Sigma-Aldrich) and observed by confocal microscopy (TCS SP5 II, Leica Microsystems, Wetzlar, Germany).

Antibodies used included rabbit anti-active-caspase3 IgG (1:100 - G7481, Promega), mouse anti-DRP1 IgG (1:100–611113, BD BioSiences), rabbit anti-FIS1 IgG (1:100 - HPA017430, Sigma-Aldrich), mouse anti- MFF IgG (1:100 – WH0055669M4, Sigma-Aldrich), mouse anti-PDI IgG (1:100 - MA3-019, ThermoFisher) and rabbit anti-TOM20 IgG (1:200 - sc-11415, Santa Cruz Biotechnology); Alexa Fluor 488/563/633 goat anti-rabbit IgG (H+L) conjugate and Alexa Fluor 488/563/633 goat anti-mouse IgG (H+L) conjugate (1:1,000, Invitrogen Molecular Probes).

### Quantitative analyses of confocal micrographs

The length and branching status of the mitochondrial network was determined by calculating the aspect ratio (AR) and end point/branch point ratio of mitochondrial particles in entire cell sections using the ImageJ 64 software according to De Vos and Sheetz^29^. The percentages of co-localisation between DRP1 and FIS1 or MFF in the entire cell sections were also assessed using ImageJ 64 software. The number of cells analysed is indicated on each column.

### Mitochondria enriched fraction preparation

RAW 264.7 cells were seeded in 6-well plates 24 h before *Brucella* infection (150,000 cells/well). At 48 h PI, cells were washed thrice with PBS and mitochondria-enriched fractions were prepared using the mammalian mitochondria isolation kit for tissue and cultured cells, according to the manufacturer’s protocol (BioVision, Milpitas, USA). These fractions were processed as described in the following section.

### Western blot analysis

RAW 264.7 cells were seeded in 6-well plates 24 h before *Brucella* infection (150,000 cells/well). At several PI time points, cells were washed three times with PBS [Tris-buffered saline (TBS) when the phosphorylated form of the protein was studied] and lysed in Radioimmunoprecipitation assay (RIPA) buffer (150 mM NaCl, 1 % NP40, 0.1 % SDS, 0.5 % DOC, 25 mM Tris; pH 7.4) supplemented with complete protease inhibitor cocktail (Roche Applied Science, Basel, Switzerland) and 4 % phosphatase inhibitor cocktail (25 mM Na_3_VO_4_, 250 mM 4-nitrophenylphosphate, 250 mM β-glycerophosphate, 125 mM NaF). After a 10 min incubation on ice, cell lysates were centrifuged for 15 min at 14 000 g at 4 °C to sediment cell debris. Lysates were then incubated for 1 h at 80 °C to inactivate remaining bacteria. Protein concentration was determined using the Pierce BCA protein assay kit (Thermo Scientific, Waltham, MA, USA). A 15 µg sample of cell lysate (2.5 µg of mitochondria-enriched fractions) was resolved by gel electrophoresis using 4–12 % bis-tris precast gels (Novex, Life Technologies, Carlsbad, CA, USA). The proteins were then electro-transferred (semi-dry device) onto a polyvinylidene fluoride (PVDF) membrane (0.45 µm) (Millipore, Billerica, MA, USA). Unspecific binding sites were blocked by incubating the membranes for 1 h at RT with the blocking solution (Li-Cor Odyssey Infrared Imaging System Blocking solution), diluted twice in PBS. Membranes were incubated overnight at 4 °C with the primary antibody and then 1 h at RT with the secondary antibody, both diluted in Li-Cor Blocking Solution supplemented with 0.1 % Tween 20. The antibodies used were the following: rabbit anti-P-IRE1 IgG (1:1,000 - ab124945, Abcam), rabbit anti-IRE1 IgG (1:1,000 - #3294, Cell Signaling), mouse anti-β-actin IgG (1:10,000 - A5441, Sigma Aldrich), mouse anti-MFN1 IgG (1:1,000 - ab57602, Abcam), mouse anti-MFN2 IgG (1:1,000 - sc-100560, Santa Cruz Biotechnology), mouse anti-OPA1 IgG (1:1,000 - BD612606, BD Biosciences), mouse anti-DRP1 IgG (1:1,000 - sc-271583, Santa Cruz Biotechnology), rabbit anti-VDAC IgG (1:1,000 - #4661, Cell Signaling), secondary antibodies coupled to infrared dyes (1:10,000, Li-Cor Biosciences, Lincoln, NE, USA).

The fluorescence intensity (detected using the Odyssey scanner) of the bands corresponding to the protein of interest was quantified using the Odyssey V3.0 application software (Li-Cor Biosciences) and normalised by the fluorescence intensity of the bands corresponding to the immunodetection of β-actin or VDAC used as loading controls.

### siRNA transfection

Silencing of DRP1, MFN1 and MFN2 expression was achieved using ONTARGETplus SMARTpool DNM1L siRNA (Dharmacon, Lafayette, CO, USA; cat. no. L012092), ON-TARGETplus SMARTpool human MFN1 (Dharmacon, cat. no. L010670) and ON-TARGET plus SMARTpool human MFN2 (Dharmacon, cat. no. L012961). Non-target siRNA (Dharmacon) was used to control for non-specific effects.

HeLa cells were seeded in 24-well plates at a density of 20,000 cells/wells 8 h before transfection and incubated at 37 °C in 5 % CO_2_. Cells were then transfected for 12 h under standard culture conditions with 20 nM siRNA using the DharmaFECT1 (Dharmacon) transfection reagent, according to the manufacturer’s instructions. The transfection media were removed and replaced with culture media for 24 h before *Brucella* infection.

### Statistical analysis

Data are reported as mean ± SD. Normality of the distributions was assessed using Shapiro-Wilk tests. Comparisons between two independent groups were performed using t tests or Mann-Whitney tests as appropriate. Comparisons of more than two groups involving one single factor were performed using one-way analysis of variance (ANOVA) or Kruskal-Wallis tests as appropriate. Comparisons between more than two groups involving two simultaneous factors were performed using two-way ANOVAs, with interaction tests between the study factors. When normality or homoscedasticity failed, ANOVAs were performed either after a Box Cox procedure^96^ correcting for heteroscedasticity (in order to facilitate interpretation, untransformed data are shown in the manuscript), or on ranked data. Pairwise comparisons after ANOVAs were performed using Holm-Sidak or Dunn’s methods. A p value < 0.05 was considered statistically significant. All calculations were performed using SigmaPlot 12.5 (Systat Software, Chicago, IL, USA) and Minitab 17.1 (Minitab Inc., State College, PA, USA) for Windows.

### Data availability

The datasets generated during the current study are available from the corresponding author on reasonable request.

## Electronic supplementary material

Supplementary Data 1

## References

[1] Gray MW, Burger G, Lang BF (1999). Mitochondrial evolution. Science.

[2] Pallen MJ (2011). Time to recognise that mitochondria are bacteria?. Trends in microbiology.

[3] Mishra P, Chan DC (2014). Mitochondrial dynamics and inheritance during cell division, development and disease. Nature reviews. Molecular cell biology.

[4] Detmer SA, Chan DC (2007). Functions and dysfunctions of mitochondrial dynamics. Nature reviews. Molecular cell biology.

[5] Youle RJ, van der Bliek AM (2012). Mitochondrial fission, fusion, and stress. Science.

[6] Szabadkai G (2004). Drp-1-dependent division of the mitochondrial network blocks intraorganellar Ca2+ waves and protects against Ca2+-mediated apoptosis. Molecular cell.

[7] West AP, Shadel GS, Ghosh S (2011). Mitochondria in innate immune responses. Nature reviews. Immunology.

[8] Cloonan SM, Choi AM (2013). Mitochondria: sensors and mediators of innate immune receptor signaling. Current opinion in microbiology.

[9] Wang C, Youle RJ (2009). The role of mitochondria in apoptosis*. Annual review of genetics.

[10] Fielden LF, Kang Y, Newton HJ, Stojanovski D (2017). Targeting mitochondria: how intravacuolar bacterial pathogens manipulate mitochondria. Cell and tissue research.

[11] Escoll P, Mondino S, Rolando M, Buchrieser C (2016). Targeting of host organelles by pathogenic bacteria: a sophisticated subversion strategy. Nature reviews. Microbiology.

[12] Khan M, Syed GH, Kim SJ, Siddiqui A (2015). Mitochondrial dynamics and viral infections: A close nexus. Biochimica et biophysica acta.

[13] Atluri VL, Xavier MN, de Jong MF, den Hartigh AB, Tsolis RM (2011). Interactions of the human pathogenic Brucella species with their hosts. Annual review of microbiology.

[14] Celli J (2015). The changing nature of the Brucella-containing vacuole. Cellular microbiology.

[15] de Jong MF, Sun YH, den Hartigh AB, van Dijl JM, Tsolis RM (2008). Identification of VceA and VceC, two members of the VjbR regulon that are translocated into macrophages by the Brucella type IV secretion system. Molecular microbiology.

[16] Taguchi Y (2015). Yip1A, a novel host factor for the activation of the IRE1 pathway of the unfolded protein response during Brucella infection. PLoS pathogens.

[17] Smith JA (2013). Brucella induces an unfolded protein response via TcpB that supports intracellular replication in macrophages. PLoS pathogens.

[18] Phillips MJ, Voeltz GK (2016). Structure and function of ER membrane contact sites with other organelles. Nature reviews. Molecular cell biology.

[19] Vannuvel K, Renard P, Raes M, Arnould T (2013). Functional and morphological impact of ER stress on mitochondria. Journal of cellular physiology.

[20] Czyz, D. M., Willett, J. W. & Crosson, S. Brucella abortus Induces a Warburg Shift in Host Metabolism That Is Linked to Enhanced Intracellular Survival of the Pathogen. *Journal of bacteriology* **199** (2017). 10.1128/JB.00227-17 PMC5512224 28559292

[21] He Y (2006). Brucella melitensis triggers time-dependent modulation of apoptosis and down-regulation of mitochondrion-associated gene expression in mouse macrophages. Infection and immunity.

[22] Gomes MT (2013). Critical role of ASC inflammasomes and bacterial type IV secretion system in caspase-1 activation and host innate resistance to Brucella abortus infection. Journal of immunology.

[23] Fugier E (2009). The glyceraldehyde-3-phosphate dehydrogenase and the small GTPase Rab 2 are crucial for Brucella replication. PLoS pathogens.

[24] Schauen M (2006). Respiratory chain deficiency slows down cell-cycle progression via reduced ROS generation and is associated with a reduction of p21CIP1/WAF1. Journal of cellular physiology.

[25] Weinberg SE, Sena LA, Chandel NS (2015). Mitochondria in the regulation of innate and adaptive immunity. Immunity.

[26] Chen Q, Vazquez EJ, Moghaddas S, Hoppel CL, Lesnefsky EJ (2003). Production of reactive oxygen species by mitochondria: central role of complex III. The Journal of biological chemistry.

[27] Trnka J, Blaikie FH, Smith RA, Murphy MP (2008). A mitochondria-targeted nitroxide is reduced to its hydroxylamine by ubiquinol in mitochondria. Free radical biology & medicine.

[28] Harbauer AB, Zahedi RP, Sickmann A, Pfanner N, Meisinger C (2014). The protein import machinery of mitochondria-a regulatory hub in metabolism, stress, and disease. Cell metabolism.

[29] De Vos KJ, Sheetz MP (2007). Visualization and quantification of mitochondrial dynamics in living animal cells. Methods in cell biology.

[30] Rappaport L, Oliviero P, Samuel JL (1998). Cytoskeleton and mitochondrial morphology and function. Molecular and cellular biochemistry.

[31] Varadi A (2004). Cytoplasmic dynein regulates the subcellular distribution of mitochondria by controlling the recruitment of the fission factor dynamin-related protein-1. Journal of cell science.

[32] Rowland AA, Voeltz GK (2012). Endoplasmic reticulum-mitochondria contacts: function of the junction. Nature reviews. Molecular cell biology.

[33] Hatch AL, Gurel PS, Higgs HN (2014). Novel roles for actin in mitochondrial fission. Journal of cell science.

[34] Stavru F, Palmer AE, Wang C, Youle RJ, Cossart P (2013). Atypical mitochondrial fission upon bacterial infection. Proceedings of the National Academy of Sciences of the United States of America.

[35] Detilleux PG, Deyoe BL, Cheville NF (1991). Effect of endocytic and metabolic inhibitors on the internalization and intracellular growth of Brucella abortus in Vero cells. American journal of veterinary research.

[36] Guzman-Verri C (2001). GTPases of the Rho subfamily are required for Brucella abortus internalization in nonprofessional phagocytes: direct activation of Cdc42. The Journal of biological chemistry.

[37] Kusumawati A (2000). Early events and implication of F-actin and annexin I associated structures in the phagocytic uptake of Brucella suis by the J-774A.1 murine cell line and human monocytes. Microbial pathogenesis.

[38] Lee JJ (2013). Toll-like receptor 4-linked Janus kinase 2 signaling contributes to internalization of Brucella abortus by macrophages. Infection and immunity.

[39] Radhakrishnan GK, Harms JS, Splitter GA (2011). Modulation of microtubule dynamics by a TIR domain protein from the intracellular pathogen Brucella melitensis. The Biochemical journal.

[40] Jeyaraju DV, Cisbani G, Pellegrini L (2009). Calcium regulation of mitochondria motility and morphology. Biochimica et biophysica acta.

[41] Hom JR, Gewandter JS, Michael L, Sheu SS, Yoon Y (2007). Thapsigargin induces biphasic fragmentation of mitochondria through calcium-mediated mitochondrial fission and apoptosis. Journal of cellular physiology.

[42] Kim DH (2012). RGS2-mediated intracellular Ca2+ level plays a key role in the intracellular replication of Brucella abortus within phagocytes. The Journal of infectious diseases.

[43] de la Fuente S, Matesanz-Isabel J, Fonteriz RI, Montero M, Alvarez J (2014). Dynamics of mitochondrial Ca2+ uptake in MICU1-knockdown cells. The Biochemical journal.

[44] Lu JR (2013). Calcium flux and calpain-mediated activation of the apoptosis-inducing factor contribute to enterovirus 71-induced apoptosis. The Journal of general virology.

[45] Xiao K, Wang Y, Chang Z, Lao Y, Chang DC (2014). p32, a novel binding partner of Mcl-1, positively regulates mitochondrial Ca(2+) uptake and apoptosis. Biochemical and biophysical research communications.

[46] Scorrano L (2013). Keeping mitochondria in shape: a matter of life and death. European journal of clinical investigation.

[47] Cui G (2014). Brucella infection inhibits macrophages apoptosis via Nedd4-dependent degradation of calpain2. Veterinary microbiology.

[48] Fernandez-Prada CM (2003). Interactions between Brucella melitensis and human phagocytes: bacterial surface O-Polysaccharide inhibits phagocytosis, bacterial killing, and subsequent host cell apoptosis. Infection and immunity.

[49] Gross A, Terraza A, Ouahrani-Bettache S, Liautard JP, Dornand J (2000). *In vitro* Brucella suis infection prevents the programmed cell death of human monocytic cells. Infection and immunity.

[50] Ma QL (2015). Brucella outer membrane protein Omp25 induces microglial cells *in vitro* to secrete inflammatory cytokines and inhibit apoptosis. International journal of clinical and experimental medicine.

[51] Scian R (2013). Brucella abortus invasion of synoviocytes inhibits apoptosis and induces bone resorption through RANKL expression. Infection and immunity.

[52] Wang M, Qureshi N, Soeurt N (2001). & Splitter, G. High levels of nitric oxide production decrease early but increase late survival of Brucella abortus in macrophages. Microbial pathogenesis.

[53] Wei P (2015). A20 promotes Brucella intracellular growth via inhibition of macrophage cell death and activation. Veterinary microbiology.

[54] Zhang K (2016). OMP31 of Brucella melitensis 16M impairs the apoptosis of macrophages triggered by TNF-alpha. Experimental and therapeutic medicine.

[55] Delpino MV, Barrionuevo P, Scian R, Fossati CA, Baldi PC (2010). Brucella-infected hepatocytes mediate potentially tissue-damaging immune responses. Journal of hepatology.

[56] Garcia Samartino C (2010). Brucella abortus induces the secretion of proinflammatory mediators from glial cells leading to astrocyte apoptosis. The American journal of pathology.

[57] Li X, He Y (2012). Caspase-2-dependent dendritic cell death, maturation, and priming of T cells in response to Brucella abortus infection. PloS one.

[58] Scian R, Barrionuevo P, Fossati CA, Giambartolomei GH, Delpino MV (2012). Brucella abortus invasion of osteoblasts inhibits bone formation. Infection and immunity.

[59] Velasquez LN (2012). Brucella abortus induces apoptosis of human T lymphocytes. Microbes and infection/Institut Pasteur.

[60] Giambartolomei GH, Arriola Benitez PC, Delpino MV (2017). Brucella and Osteoarticular Cell Activation: Partners in Crime. Frontiers in microbiology.

[61] Baldi PC, Giambartolomei GH (2013). Immunopathology of Brucella infection. Recent patents on anti-infective drug discovery.

[62] Pham CG (2004). Ferritin heavy chain upregulation by NF-kappaB inhibits TNFalpha-induced apoptosis by suppressing reactive oxygen species. Cell.

[63] Xavier MN (2013). PPARgamma-mediated increase in glucose availability sustains chronic Brucella abortus infection in alternatively activated macrophages. Cell host & microbe.

[64] Shi L (2015). Infection with Mycobacterium tuberculosis induces the Warburg effect in mouse lungs. Scientific reports.

[65] Li T (2016). Brucella Melitensis 16M Regulates the Effect of AIR Domain on Inflammatory Factors, Autophagy, and Apoptosis in Mouse Macrophage through the ROS Signaling Pathway. PloS one.

[66] Jin OU (2013). Overexpression of ryanodine receptor type 1 enhances mitochondrial fragmentation and Ca2+-induced ATP production in cardiac H9c2 myoblasts. American journal of physiology. Heart and circulatory physiology.

[67] Yu T, Jhun BS, Yoon Y (2011). High-glucose stimulation increases reactive oxygen species production through the calcium and mitogen-activated protein kinase-mediated activation of mitochondrial fission. Antioxidants & redox signaling.

[68] Stavru F, Bouillaud F, Sartori A, Ricquier D, Cossart P (2011). Listeria monocytogenes transiently alters mitochondrial dynamics during infection. Proceedings of the National Academy of Sciences of the United States of America.

[69] Park J (2015). Loss of mitofusin 2 links beta-amyloid-mediated mitochondrial fragmentation and Cdk5-induced oxidative stress in neuron cells. Journal of neurochemistry.

[70] Hirata N, Yamada S, Asanagi M, Sekino Y, Kanda Y (2016). Nicotine induces mitochondrial fission through mitofusin degradation in human multipotent embryonic carcinoma cells. Biochemical and biophysical research communications.

[71] Chen Y (2013). Mitofusin 2 protects hepatocyte mitochondrial function from damage induced by GCDCA. PloS one.

[72] Malhotra A, Dey A, Prasad N, Kenney AM (2016). Sonic Hedgehog Signaling Drives Mitochondrial Fragmentation by Suppressing Mitofusins in Cerebellar Granule Neuron Precursors and Medulloblastoma. Molecular cancer research: MCR.

[73] Ke Y, Wang Y, Li W, Chen Z (2015). Type IV secretion system of Brucella spp. and its effectors. Frontiers in cellular and infection microbiology.

[74] Smith, E. P., Miller, C. N., Child, R., Cundiff, J. A. & Celli, J. Postreplication Roles of the Brucella VirB Type IV Secretion System Uncovered via Conditional Expression of the VirB11 ATPase. *mBio* **7** (2016). 10.1128/mBio.01730-16 PMC5137499 27899503

[75] Jain P, Luo ZQ, Blanke SR (2011). Helicobacter pylori vacuolating cytotoxin A (VacA) engages the mitochondrial fission machinery to induce host cell death. Proceedings of the National Academy of Sciences of the United States of America.

[76] Escoll, P. *et al*. Legionella pneumophila Modulates Mitochondrial Dynamics to Trigger Metabolic Repurposing of Infected Macrophages. Cell host & microbe **22**, 302–316 e307 (2017). 10.1016/j.chom.2017.07.020 28867389

[77] Archambaud C (2010). Contrasting roles of macrophages and dendritic cells in controlling initial pulmonary Brucella infection. European journal of immunology.

[78] Barbier T (2017). Erythritol Availability in Bovine, Murine and Human Models Highlights a Potential Role for the Host Aldose Reductase during BrucellaInfection. Frontiers in microbiology.

[79] Anderson TD, Cheville NF (1986). Ultrastructural morphometric analysis of Brucella abortus-infected trophoblasts in experimental placentitis. Bacterial replication occurs in rough endoplasmic reticulum. The American journal of pathology.

[80] Cha SB (2013). Early transcriptional responses of internalization defective Brucella abortus mutants in professional phagocytes, RAW 264.7. BMC genomics.

[81] Delpino MV, Fossati CA, Baldi PC (2009). Proinflammatory response of human osteoblastic cell lines and osteoblast-monocyte interaction upon infection with Brucella spp. Infection and immunity.

[82] Pesce Viglietti AI (2015). Brucella abortus Invasion of Osteocytes Modulates Connexin 43 and Integrin Expression and Induces Osteoclastogenesis via Receptor Activator of NF-kappaB Ligand and Tumor Necrosis Factor Alpha Secretion. Infection and immunity.

[83] Aggarwal BB (2003). Signalling pathways of the TNF superfamily: a double-edged sword. Nature reviews. Immunology.

[84] Mattson MP (2008). Awareness of hormesis will enhance future research in basic and applied neuroscience. Critical reviews in toxicology.

[85] Suzuki M, Danilchanka O, Mekalanos JJ (2014). Vibrio cholerae T3SS Effector VopE Modulates Mitochondrial Dynamics and Innate Immune Signaling by Targeting Miro GTPases. Cell host & microbe.

[86] Van Laar VS, Berman SB (2009). Mitochondrial dynamics in Parkinson’s disease. Experimental neurology.

[87] Zhu X, Perry G, Smith MA, Wang X (2013). Abnormal mitochondrial dynamics in the pathogenesis of Alzheimer’s disease. Journal of Alzheimer’s disease: JAD.

[88] Yoon Y, Galloway CA, Jhun BS, Yu T (2011). Mitochondrial dynamics in diabetes. Antioxidants & redox signaling.

[89] Liesa M, Palacin M, Zorzano A (2009). Mitochondrial dynamics in mammalian health and disease. Physiological reviews.

[90] Wai T, Langer T (2016). Mitochondrial Dynamics and Metabolic Regulation. Trends in endocrinology and metabolism: TEM.

[91] Santos D, Esteves AR, Silva DF, Januario C, Cardoso SM (2015). The Impact of Mitochondrial Fusion and Fission Modulation in Sporadic Parkinson’s Disease. Molecular neurobiology.

[92] Papanicolaou KN (2012). Cardiomyocyte deletion of mitofusin-1 leads to mitochondrial fragmentation and improves tolerance to ROS-induced mitochondrial dysfunction and cell death. American journal of physiology. Heart and circulatory physiology.

[93] Khacho M (2016). Mitochondrial Dynamics Impacts Stem Cell Identity and Fate Decisions by Regulating a Nuclear Transcriptional Program. Cell stem cell.

[94] Pireaux V (2016). Myeloperoxidase-Oxidized LDLs Enhance an Anti-Inflammatory M2 and Antioxidant Phenotype in Murine Macrophages. Mediators of inflammation.

[95] de Barsy M (2011). Identification of a Brucella spp. secreted effector specifically interacting with human small GTPase Rab2. Cellular microbiology.

[96] Box GEP, Cox DR (1964). An Analysis of Transformations. Journal of the Royal Statistical Society.

