# Supplementary material for: Mitochondrial fragmentation affects neither the sensitivity to TNFα-induced apoptosis of Brucella-infected cells nor the intracellular replication of the bacteria
Source: Sci Rep. 2018 Mar 26;8:5173. doi: 10.1038/s41598-018-23483-3 (PMC5979954; doi:10.1038/s41598-018-23483-3)
Supplement: Supplementary file 1 — Supplementary Data 1 [file 41598_2018_23483_MOESM1_ESM.pdf]

SUPPLEMENTARY DATA

Mitochondrial fragmentation affects neither the sensitivity to TNFα-induced apoptosis of *Brucella*-infected cells nor the intracellular replication of the bacteria

Elodie Lobet[1,] Kevin Willemart[2], Noëlle Ninane[1], Catherine Demazy[1], Jaroslaw Sedzicki[3], Christophe Lelubre[4], Xavier De Bolle[2,] Patricia Renard[1], Martine Raes[1], Christoph Dehio[3], Jean-Jacques Letesson[2#], Thierry Arnould[1#*]

1. Laboratory of Biochemistry and Cell Biology (URBC, Unité de Recherche en Biologie Cellulaire)-NARILIS (Namur Research Institute for Life Sciences), University of Namur, Rue de Bruxelles 61, 5000 Namur, Belgium
2. Microorganisms Biology Research Unit (URBM, Unité de Recherche en Biologie des Microorganismes), University of Namur, Rue de Bruxelles 61, 5000 Namur, Belgium
3. Biozentrum, University of Basel, Klingelbergstrasse 50 / 70, 4056 Basel, Switzerland
4. Laboratory of Experimental Medicine (ULB 222 Unit), Medicine Faculty, Université Libre de Bruxelles, CHU de Charleroi, Rue de Gozée 706, 6110 Montigny-le-Tilleul, Belgium.

# : co-last senior authors
*: corresponding author

SUPPLEMENTARY METHODS

Cell culture

BeWo cells (ATCC) were cultured in F12K medium (Gibco) supplemented with 10% FBS (Gibco).

Bacterial strains

Brucella melitensis 16M mCherry is a CO_2-independent virulent smooth strain that constitutively expresses fluorescent mCherry due to the integration of a plasmid containing the mCherry coding sequence and a kanamycin resistance marker[96]. Cultures of *Brucella* were freshly inoculated from frozen stock into 2YT medium [1% yeast extract (Invitrogen), 1.6% bactotryptone (Invitrogen), 0.5% NaCl (Invitrogen)] plates supplemented with 10 µg/ml kanamycin (AppliChem Panreact), before subcultures were obtained in 2YT broth media (aerobic condition, 37°C).

Cellular infection

The same procedure was used as described in the manuscript (BeWo: MOI 100).

Immunostaining

The same procedure was used as described in the manuscript. An additional antibody was mouse IgG anti-α-tubulin (1:1,000 – T5168, Sigma-Aldrich).
For the COX I and COX IV immunostaining, cells were fixed by incubation for 15 min in an ice cold solution of 80% methanol and 20% acetone. The subsequent steps of the procedure were as described in the manuscript. Antibodies were mouse anti-COX I IgG (1:100 - 1D6E1A8, Abcam) and mouse anti-COX IV IgG (1:100 – 20E8C12, Abcam).

ATP Content

RAW 264.7 cells were seeded in 12-well plates at a density of 50,000 cells/well. After 24 h, cells were incubated in the presence or absence of 10 nM myxothiazol or 100 nM antimycin A for different periods. ATP content was measured using a luciferin–luciferase reaction assay. Cells were permeabilised for 10 s with 500 µl of ATP-releasing agent (Sigma–Aldrich). The solution was recovered and diluted from 50 to 300 times (according to the time of incubation) in pure water before incubation with an ATP assay mix solution (Sigma–Aldrich) at a 1:1 (v/v) ratio. Relative light units (RLU), based on emitted photons, were quantified using a luminometer (FB12 Luminometer; Berthold Detection Systems), and results were normalised for protein content determined using the Folin assay[97].

mtROS content

The relative production of mitochondrial superoxide anion radicals was measured in RAW 264.7 cells incubated with or without 100 nM antimycin A for different periods. At the end of the incubations, cells were loaded for 20 min at 37 °C with 5 µM MitoSOX Red fluorescent specific dye (Molecular Probes, Life Technologies) diluted in complete Hanks's balanced salt solution (HBSS) buffer (0.137 M NaCl, 5.4 mM KCl, 0.25 mM Na_2HPO_4, 0.44 mM KH_2PO_4, 4.2 mM $NaHCO_3$; pH 7.4, 1.3 mM $CaCl_2$, 1.0 mM $MgSO_4$,). Cells were then washed with PBS, gently scraped, centrifuged and resuspended in 500 µl of HBSS buffer. Cells were analysed by flow cytometry with a FACSCalibur instrument (BD Biosciences), using the FL2-H channel, and data were processed using the BD CellQuest Pro software.

Western blot analysis

HeLa cells were seeded in 6-well plates 8 h before siRNA transfection (100,000 cells/well). At several times post-transfection, cells were washed three times with PBS and lysed in DLA

buffer (7 M urea, 2 M thiourea, 1% CHAPS, 1% ASB14, 1% SDS, 30 mM Tris; pH 8.5) supplemented with a complete protease inhibitor cocktail (Roche) and 4% phosphatase inhibitor cocktail (25 mM Na_3VO_4, 250 mM 4-nitrophenylphosphate, 250 mM β-glycerophosphate, 125 mM NaF). After a 10 min incubation on ice, cell lysates were sonicated to degrade the chromatin and centrifuged for 15 min at 14 000 g at 4 °C to sediment cell debris. Protein concentration was determined using the 660 nm Pierce assay kit (Thermo Fisher Scientific). A 10 µg sample of cell lysate (2.5 µg of mitochondria-enriched fraction) was resolved by gel electrophoresis using 10% bis-tris precast gels (Novex, Life Technologies). The proteins were then electro-transferred (semi-dry device) onto a polyvinylidene fluoride (PVDF) membrane (0.45 µm) (Millipore). Unspecific binding sites were blocked by incubating the membranes for 1 h at RT with the blocking solution (Li-Cor Odyssey Infrared Imaging System Blocking solution), diluted twice in PBS. Membrane were incubated overnight at 4 °C with the primary antibody and then for 1 h at RT with the secondary antibody, both diluted in Li-Cor Blocking Solution supplemented with 0.1% Tween 20. Antibodies used are the following: β-actin (1:10,000 - A5441, Sigma Aldrich), MFN1 (1:1,000 - 13798-1-AP, ProteinTech), MFN2 (1:1,000 - sc-100560, Santa Cruz), DRP1 (1:1,000 - #8570, Cell Signaling), secondary antibodies coupled to infrared dyes (1:10,000, Li-Cor Biosciences).

The fluorescence intensity (detected using an Odyssey scanner) of the bands corresponding to the protein of interest was quantified using the Odyssey V3.0 application software (Li-Cor Biosciences) and normalised for the fluorescence intensity signal of the bands corresponding the immunodetection of β-actin used as loading controls..

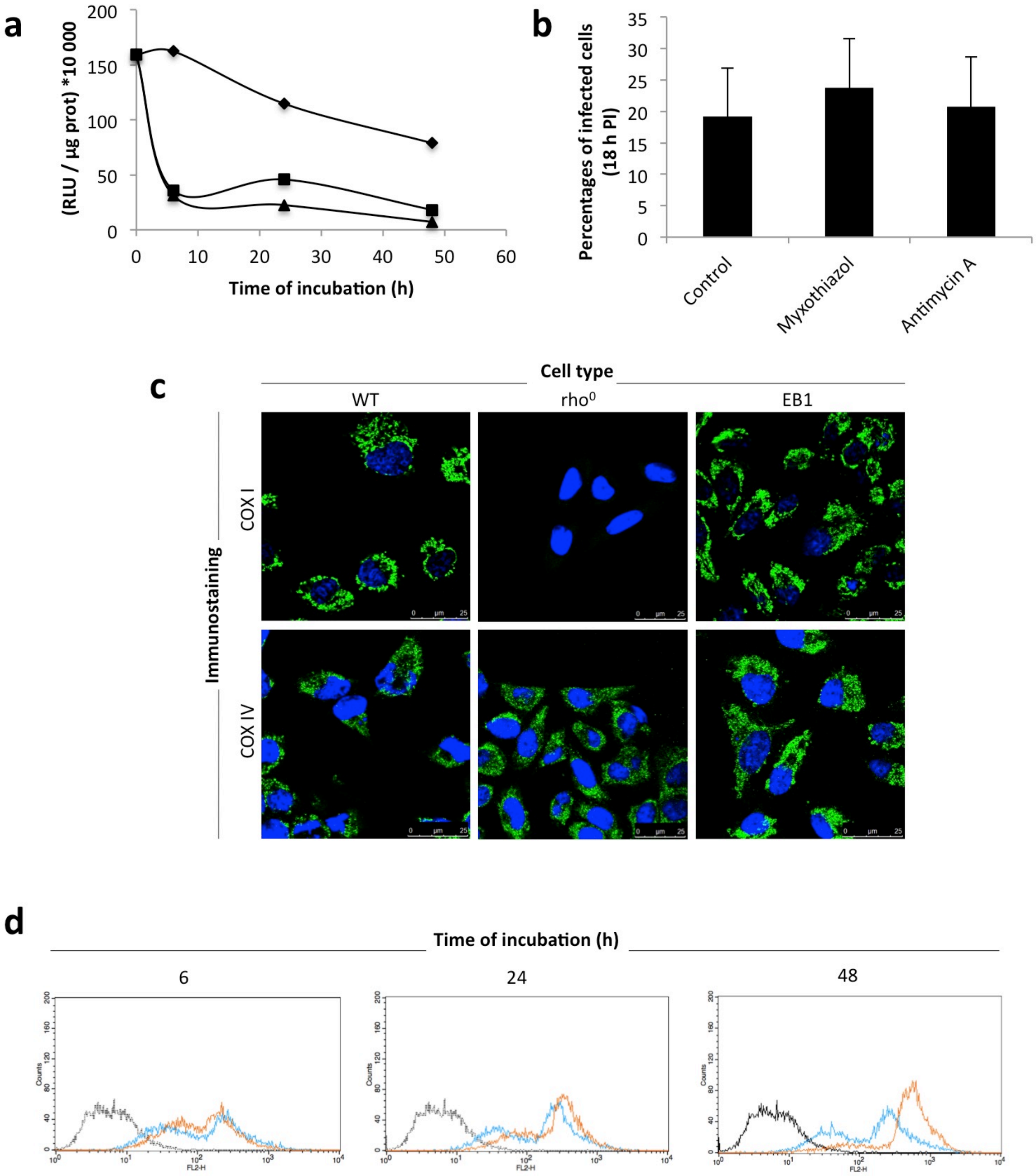

Figure S1: Mitochondrial dysfunction – characterization and impact on *Brucella* replication in RAW 264.7 cells

a. ATP content in RAW 264.7 cells incubated or not (diamonds) with 10 nM myxothiazol (squares) or 100 nM antimycin A (triangles). (n=2)

b. Percentage of infected RAW 264.7 cells pre-incubated or not (control) for 6 h with 10 nM myxothiazol or 100 nM antimycin A and infected with *B. abortus* 2308 mCherry with or without inhibitor – analysis performed at 18 h PI. The percentages of infected cells were determined by counting mCherry positive cells by confocal microscopy among, at least, 10 000 cells taken from selected microscopic fields. Results represent means ± SD for three independent experiments (n=3). Statistical analysis: one-way ANOVA on Ranks (Kruskal-Wallis).

c. COX I and COX IV immunostaining in HeLa WT, EB1 and rho^0. (n=1) Blue: Nuclei (TO-PRO3) / Green: COX I/COX IV (Alexa488).

d. mtROS content of RAW 264.7 cells incubated or not (blue) with 100 nM antimycin A (orange), assessed by flow cytometry. Black lines correspond to unlabelled cells used as control versus MitoSox Red labelled cells. (n=1)

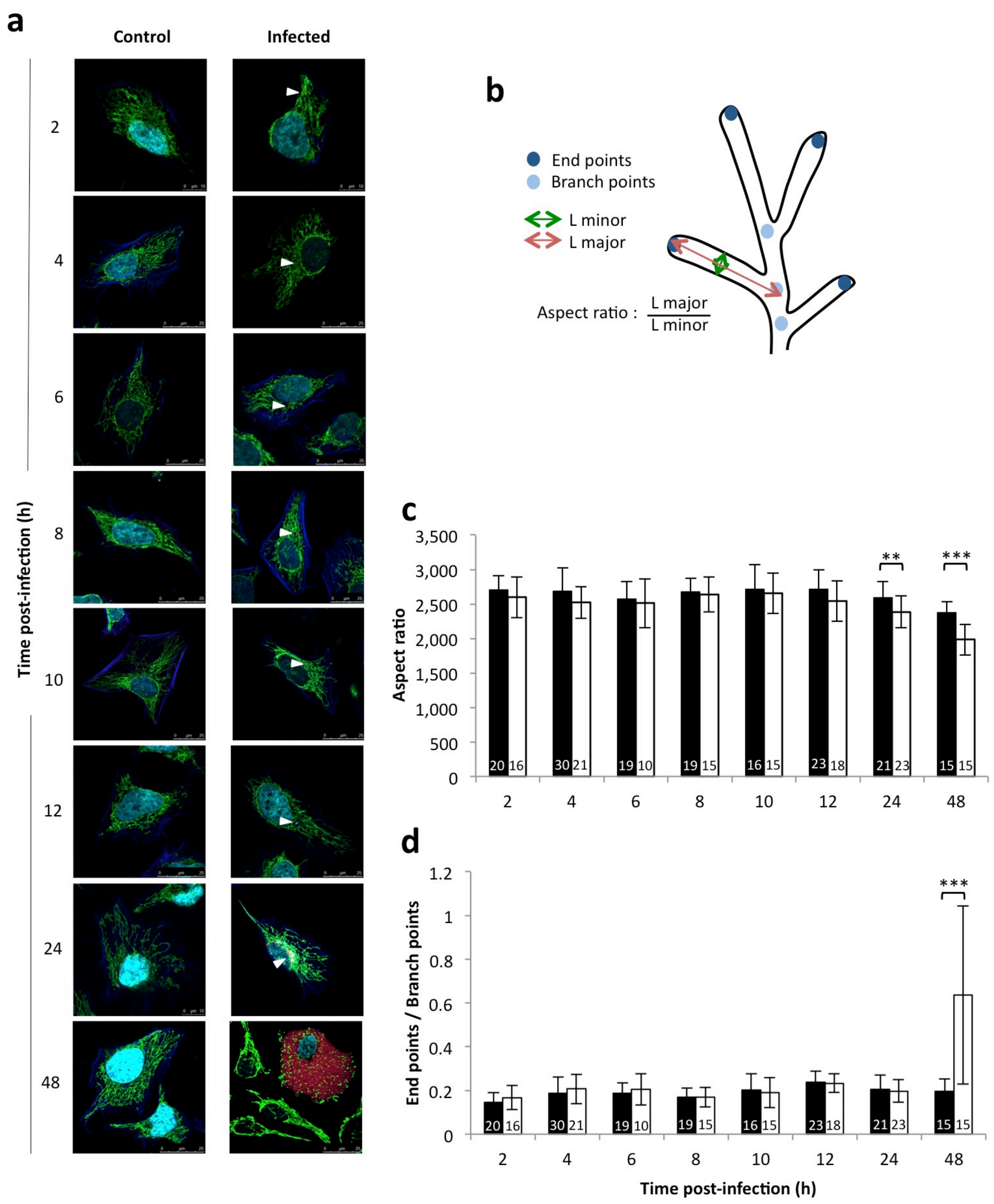

Figure S2: *Brucella abortus* infection induces mitochondrial fragmentation in infected HeLa cells: complete kinetics

a. TOM20 immunostaining in HeLa cells infected or not (control) with *B. abortus* 2308 mCherry at different times post-infection from 2 to 48 h PI. (n=1) Green: TOM20 (Alexa488) / Red: *B. abortus* 2308 (mCherry) / Blue: Actin (Phalloïdin633) / Turquoise: Nuclei (Hoechst). White arrows point the bacteria inside the infected cells.

b. Quantification of mitochondrial morphology using ImageJ software. Measure of the network connexion: end point/branch point ratio. Measure of the network elongation: aspect ratio.

c./d. Aspect ratio (c) and end point/branch point ratio (d) of HeLa cells infected (white) or not (black) with *B. abortus* 2308 mCherry at different times post-infection from 2 to 48 h PI. (n=1) Statistical analysis: Rank sum Test (Mann-Whitney). (**: $P < 0.01$, ***: $P < 0.001$). The numbers indicated in the columns represent the number of cells analysed for each condition.

a

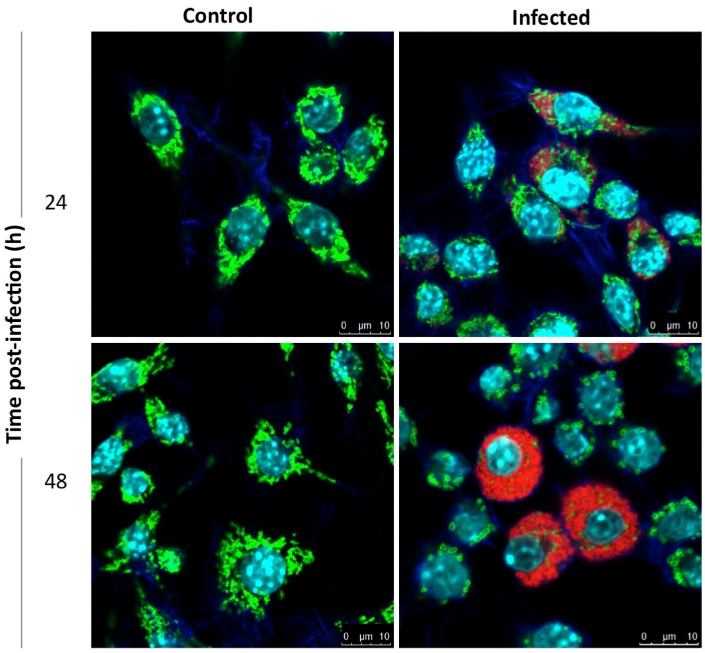

b

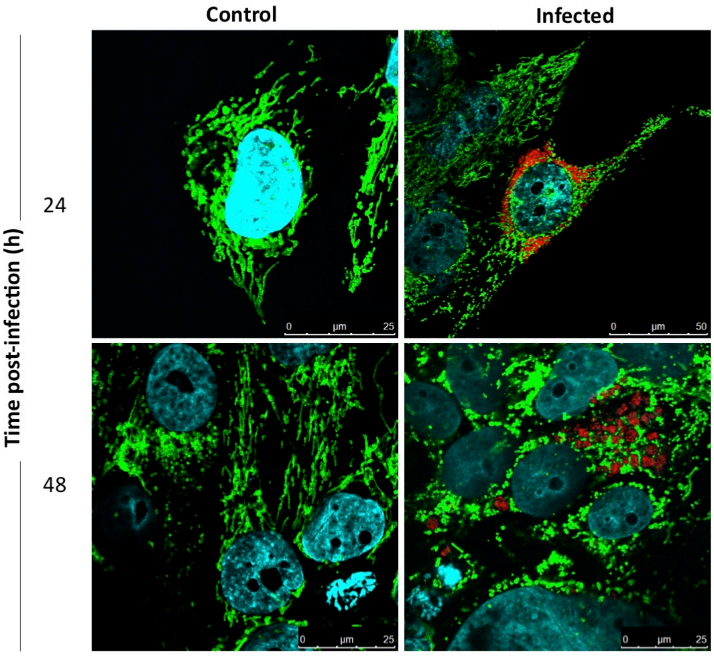

Figure S3: *Brucella abortus* infection induces mitochondrial fragmentation in infected RAW 264.7 and BeWo cells

a. TOM20 immunostaining in RAW 264.7 macrophages infected or not (control) with *B. abortus* 2308 mCherry - 2 to 48 h PI. (n=1) Green: TOM20 (Alexa488) / Red: *B. abortus* 2308 (mCherry) / Blue: Actin (Phalloïdin633) / Turquoise: Nuclei (Hoechst)

b. TOM20 immunostaining in BeWo cells infected or not (control) with *B. abortus* 2308 mCherry - 48 h PI. (Representative of n=3) Green: TOM20 (Alexa488) / Red: *B. abortus* 2308 (mCherry) / Turquoise: Nuclei (Hoechst)

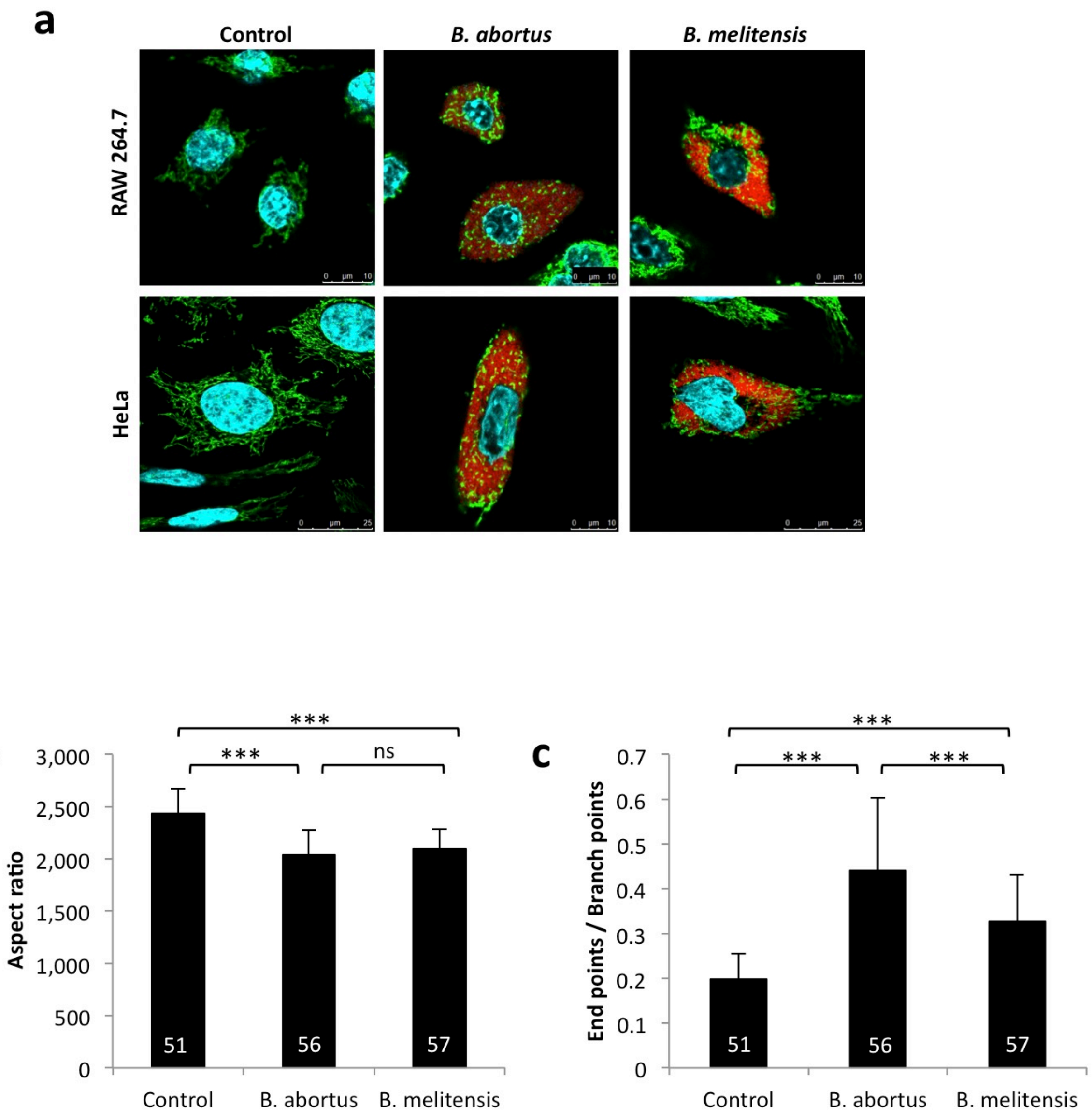

Figure S4: *Brucella melitensis* infection also induces mitochondrial fragmentation

a. TOM20 immunostaining in RAW 264.7 and HeLa cells infected or not (control) with *B. abortus* 2308 mCherry or *B. melitensis* 16M mCherry at 48 h PI. (Representative of n=3) Green: TOM20 (Alexa488) / Red: *B. abortus* (mCherry) / Turquoise: Nuclei (Hoechst)

b./c. Aspect ratio (b) and end point/branch point ratio (c) of HeLa cells infected or not (control) with *B. abortus* 2308 mCherry or *B. melitensis* 16M mCherry. Results represent means ± SD for three independent experiments (n=3). Statistical analysis: one-way ANOVA on Ranks (Kruskal-Wallis). (***: $P < 0.001$). The numbers indicated in the columns represent the number of cells analysed for each condition.

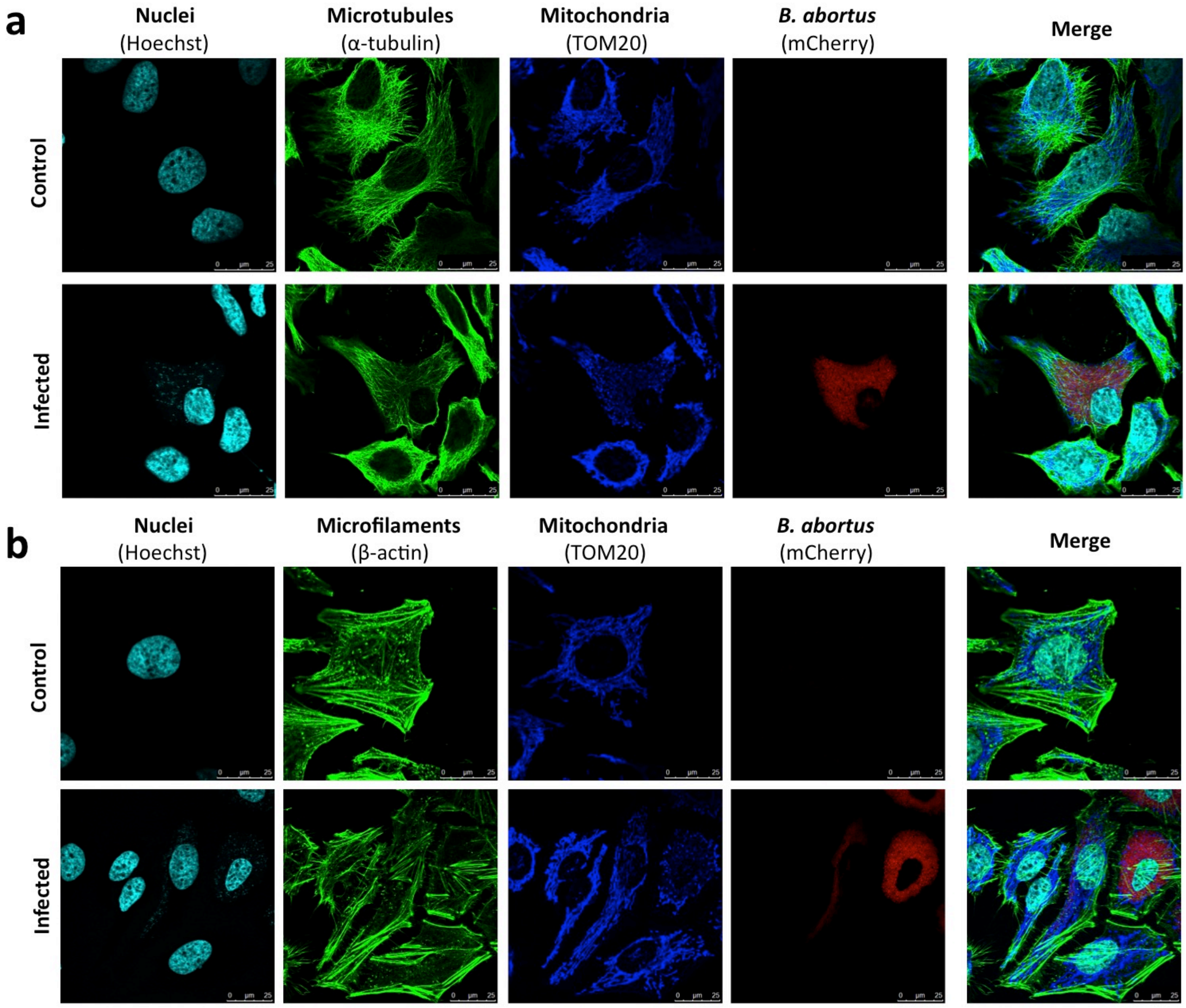

Figure S5: ***Brucella abortus*** **infection does not alter the organisation of actin and tubulin cytoskeletons**

a. TOM20 and α-tubulin co-immunostaining in HeLa cells infected or not (control) with *B. abortus* 2308 mCherry (48 h PI). (n=1) Green: α-tubulin (Alexa488) / Red: *B. abortus* 2308 (mCherry) / Blue: TOM20 (Alexa633) / Turquoise: Nuclei (Hoechst)

b. TOM20 and actin (immuno)staining in HeLa cells infected or not (control) with *B. abortus* 2308 mCherry (48 h PI). (n=1) Green: Actin (Phalloidin488) / Red: *B. abortus 2308* (mCherry) / Blue: TOM20 (Alexa633) / Turquoise: Nuclei (Hoechst)

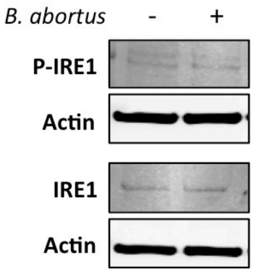

Figure S6: UPR is not responsible for *Brucella abortus*-induced mitochondrial fragmentation
Western blot analysis of P-IRE1 and IRE1 abundance in HeLa cells infected or not with *B. abortus* 2308 mCherry - 48 h PI. Actin abundance was assessed on the same blot as a loading control. (Representative of n=3)

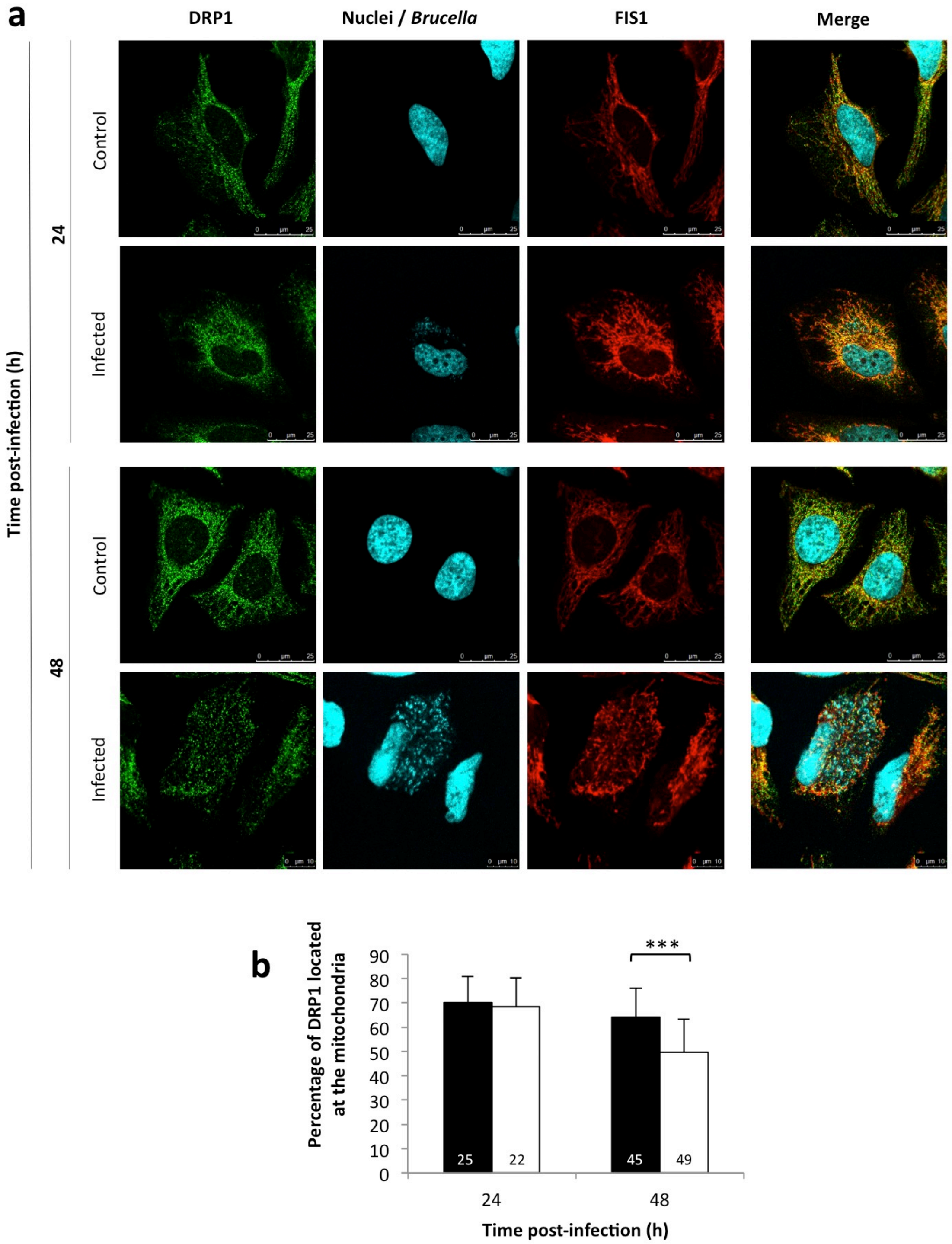

Figure S7: *Brucella abortus* infection does not induce mitochondrial recruitment of DRP1 by FIS1
a. FIS1 and DRP1 co-immunostaining in HeLa cells infected or not (control) with *B. abortus* 2308 (24 and 48 h PI). (Representative of n=3) Green: DRP1 (Alexa488) / Red: FIS1 (Alexa563) / Turquoise: Nuclei and *B. abortus* 2308 (Hoechst)
b. Quantification of the percentage of DRP1 co-localising with FIS1 in HeLa cells infected (white) or not (black) with *B. abortus* 2308**.** Results represent means ± SD for three independent experiments (n=3). Statistical analysis: Rank sum test (Mann-Whitney). (*: $P < 0.05$, **: $P < 0.01$, ***: $P < 0.001$) The numbers indicated in the columns represent the number of cells analysed for each condition.

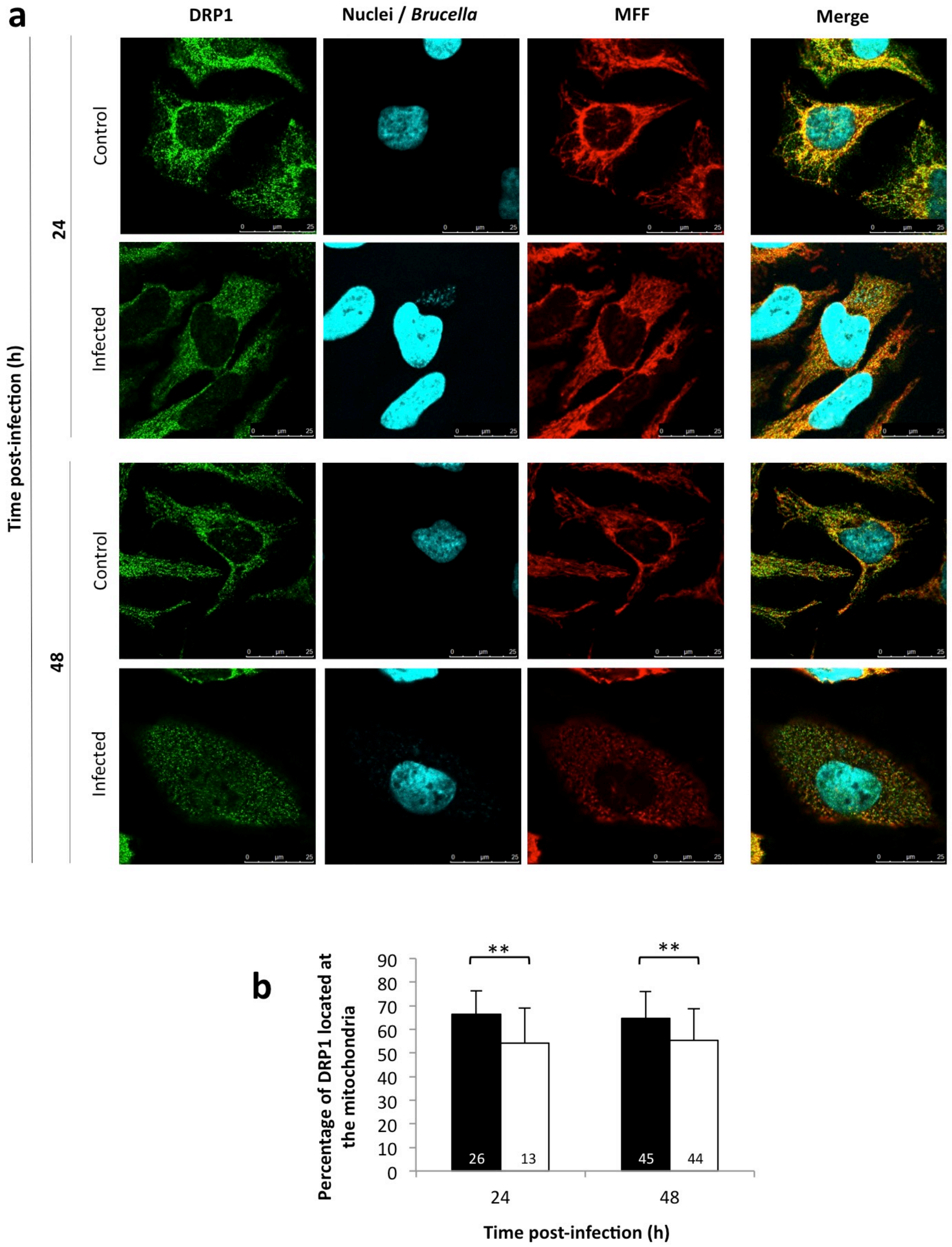

Figure S8: *Brucella abortus* infection does not induce mitochondrial recruitment of DRP1 by MFF

a. MFF and DRP1 co-immunostaining in HeLa cells infected or not (control) with *B. abortus* 2308 (24 and 48 h PI). (Representative of n=3) Green: DRP1 (Alexa488) / Red: MFF (Alexa563) / Turquoise: Nuclei and *B. abortus* 2308 (Hoechst)

b. Quantification of the percentage of DRP1 co-localising with MFF in HeLa cells infected (white) or not (black) with *B. abortus* 2308**.** Results represent means ± SD for three independent experiments (n=3). Statistical analysis: Rank sum test (Mann-Whitney). (*: $P < 0.05$, **: $P < 0.01$, ***: $P < 0.001$) The numbers indicated in the columns represent the number of cells analysed for each condition.

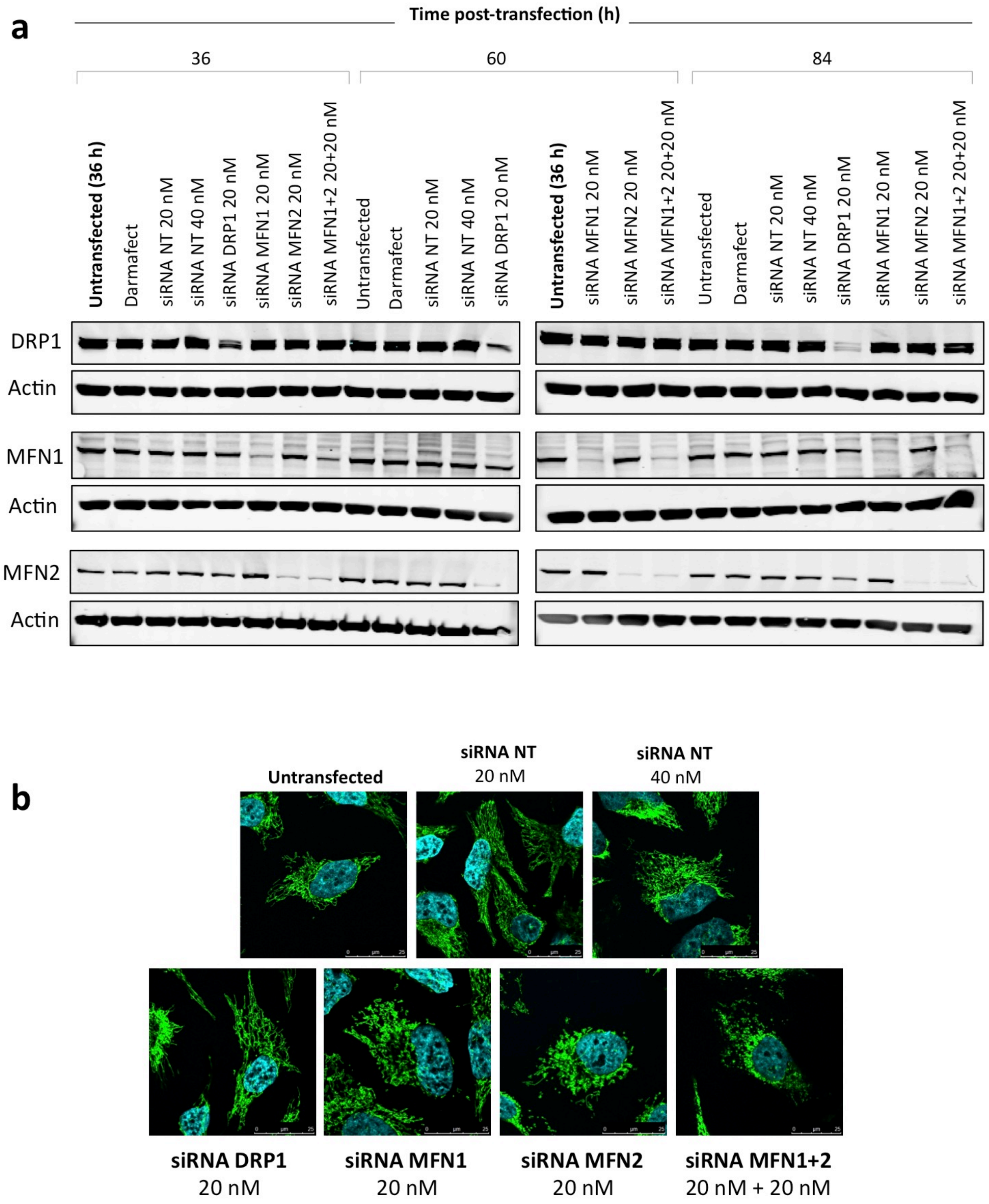

Figure S9: Modulation of the mitochondrial morphology using siRNA

a. DRP1, MFN1 and MFN2 abundance in HeLa cells transfected or not transfected (untransfected) with non-target siRNA or siRNAs against DRP1, MFN1, MFN2 or MFN1/2 and analysed by western blotting at several time points post-transfection. Actin abundance was assessed on the same blot as a loading control. (n=1)

b. TOM20 immunostaining in HeLa cells transfected or not with siRNA non target siRNA or siRNA against DRP1, MFN1, MFN2 or MFN1/2 at 36 h post-transfection (corresponding to the day of infection) (n=1)

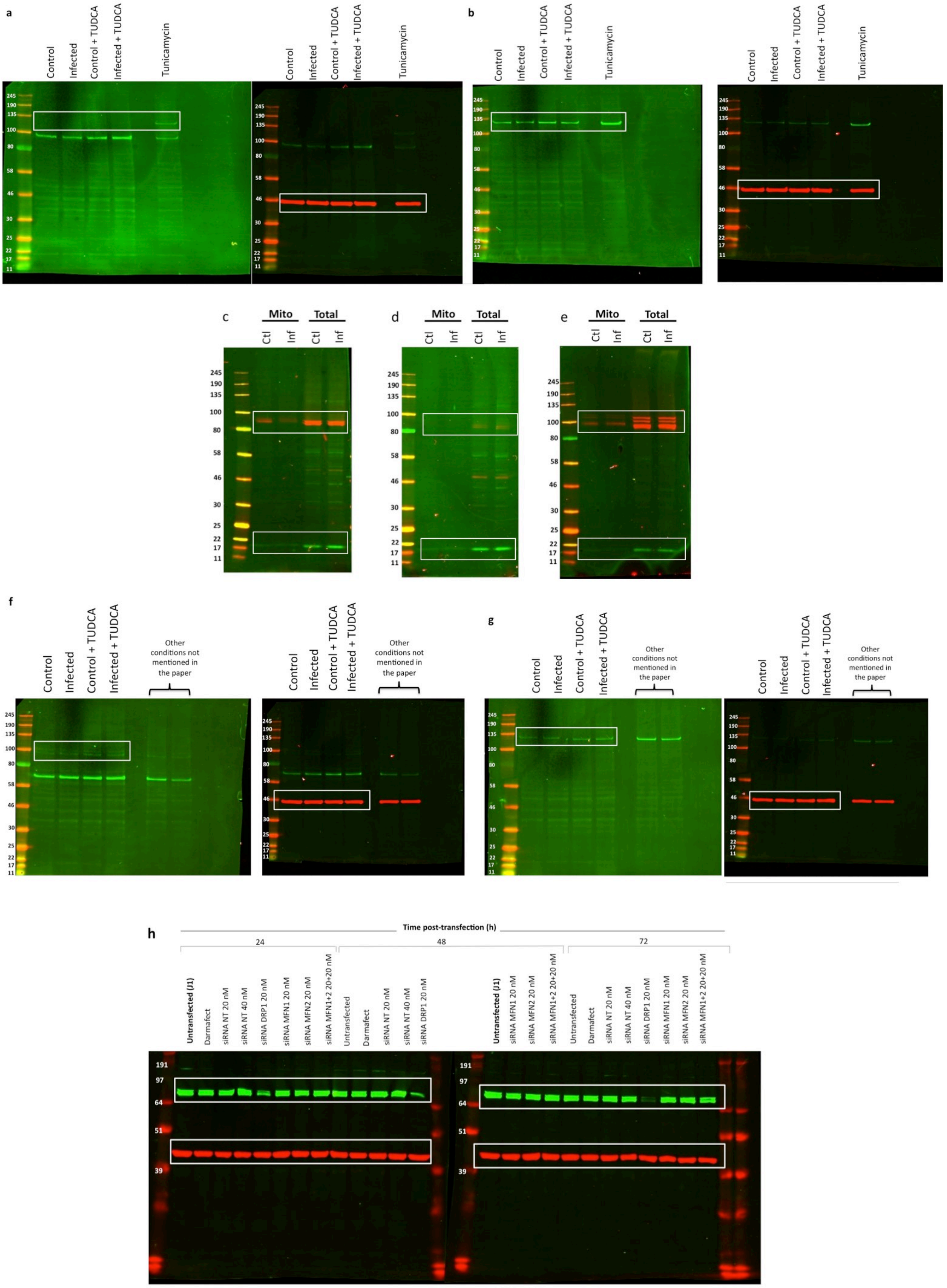
a
Control
Infected
Control + TUDCA
Infected + TUDCA
Tunicamycin
b
c
d
e
Mito
Total
Ctl
Inf
f
g
Other conditions not mentioned in the paper
h
Time post-transfection (h)
24
48
72
Untransfected (J1)
Darmafect
siRNA NT 20 nM
siRNA NT 40 nM
siRNA DRP1 20 nM
siRNA MFN1 20 nM
siRNA MFN2 20 nM
siRNA MFN1+2 20+20 nM
Untransfected

i

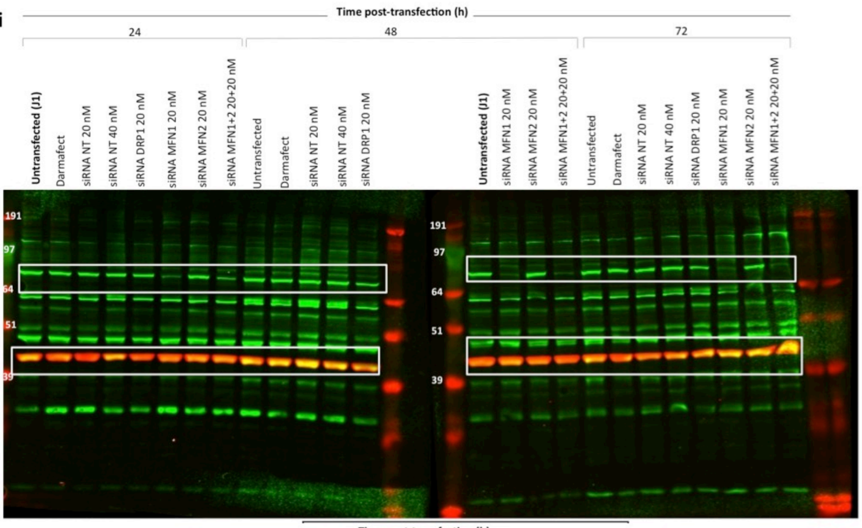

j

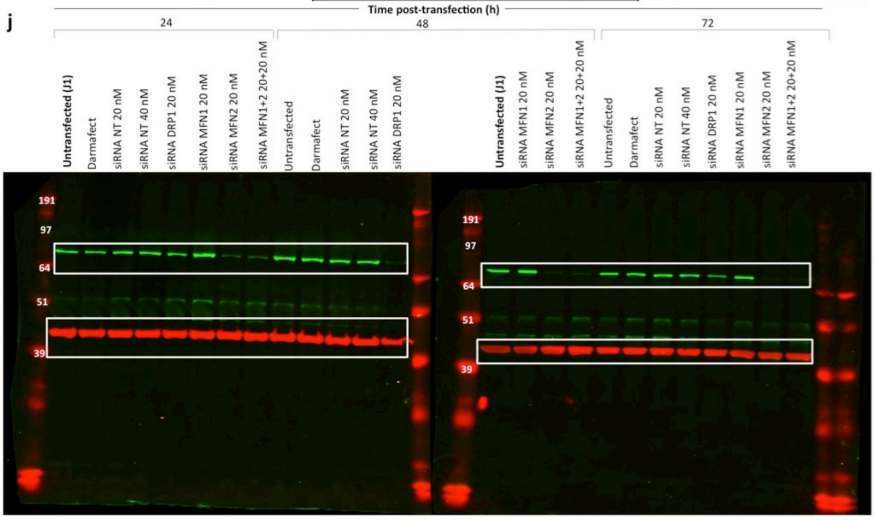

Figure S10: Complete blots for the crop blot presented in figures 4, 5, S6 and S9.
Protein immunostaining have been performed with secondary antibodies coupled to infrared dyes (IRDye) detected using the Odyssey scanner (Li-Cor Bioscences). Green: 800 nm IRDye / Red: 680 nm IRDye
White frames indicate cropped zones presented in the manuscript figures.

a. **Figure 4e** The two panels represent the same blot scanned at different intensities. Left blot: P-IRE1 (800 nm IRDye - Scan intensity: 6) – Right blot: P-IRE (800 nm IRDye - scan intensity: 5) and Actin (680 nm IRDye - scan intensity: 2)
b. **Figure 4e** The two panels represent the same blot scanned at different intensities. Left blot: IRE1 (800 nm IRDye - Scan intensity: 6) – Right blot: IRE (800 nm IRDye - scan intensity: 5) and Actin (680 nm IRDye - scan intensity: 2)
c. **Figure 5h:** MFN1 (800 nm IRDye - Scan intensity: 5) / VDAC (680 nm IRDye - Scan intensity: 4)
d. **Figure 5h**: MFN2 (800 nm IRDye - Scan intensity: 5) / VDAC (680 nm IRDye - Scan intensity: 5)
e. **Figure 5h**: OPA1 (800 nm IRDye - Scan intensity: 5) / VDAC (680 nm IRDye - Scan intensity: 4)
f. **Figure S6b** The two panels represent the same blot scanned at different intensities. Left blot: P-IRE1 (800 nm IRDye - Scan intensity: 6) – Right blot: P-IRE (800 nm IRDye - scan intensity: 5) and Actin (680 nm IRDye - scan intensity: 2)
g. **Figure S6b** The two panels represent the same blot scanned at different intensities. Left blot: IRE1 (800 nm IRDye - Scan intensity: 6) – Right blot: IRE (800 nm IRDye - scan intensity: 5) and Actin (680 nm IRDye - scan intensity: 2)
h. **Figure S9a**: DRP1 (800 nm IRDye - Scan intensity: 5) / Actin (680 nm IRDye - Scan intensity: 2)
i. **Figure S9a:** MFN1 (800 nm IRDye- Scan intensity: 5) / Actin (680 nm IRDye - Scan intensity: 2)
j. **Figure S9a**: MFN2 (800 nm IRDye - Scan intensity: 5) / Actin (680 nm IRDye - Scan intensity: 2)
